# Heteronuclear Complexes with Promising Anticancer Activity against Colon Cancer

**DOI:** 10.3390/biomedicines12081763

**Published:** 2024-08-05

**Authors:** Elena Atrián-Blasco, Javier Sáez, Maria Jesús Rodriguez-Yoldi, Elena Cerrada

**Affiliations:** 1 Departamento de Química Inorgánica, Instituto de Síntesis Química y Catálisis Homogénea—ISQCH, Consejo Superior de Investigaciones Científicas, Universidad de Zaragoza, 50009 Zaragoza, Spainj.saez@unizar.es (J.S.); 2 Departamento de Farmacología y Fisiología, Medicina Legal y Forense, Unidad de Fisiología, Facultad de Veterinaria, Ciber de Fisiopatología de la Obesidad y Nutrición (CIBERobn), Instituto Agroalimentario de Aragón (IA2), 50013 Zaragoza, Spain; mjrodyol@unizar.es; 3 Instituto de Investigación Sanitaria de Aragón (IIS Aragón), 50009 Zaragoza, Spain

**Keywords:** heteronuclear complexes, gold, copper, zinc, thioredoxin reductase, ROS, BSA, DNA

## Abstract

This study investigates the activity of novel gold(I) and copper(I)/zinc(II) heteronuclear complexes against colon cancer. The synthesised heteronuclear Au(I)-Cu(I) and Au(I)-Zn(II) complexes were characterised and evaluated for their anticancer activity using human colon cancer cell lines (Caco-2). The complexes exhibited potent cytotoxicity, with IC_50_ values in the low micromolar range, and effectively induced apoptosis in cancer cells. In the case of complex [Cu{Au(Spy)(PTA)}_2_]PF_6_ (**2**), its cytotoxicity is ×10 higher than its mononuclear precursor, while showing low cytotoxicity towards differentiated healthy cells. Mechanistic studies revealed that complex **2** inhibits the activity of thioredoxin reductase, a key enzyme involved in redox regulation, leading to an increase in reactive oxygen species (ROS) levels and oxidative stress, in addition to an alteration in DNA’s tertiary structure. Furthermore, the complexes demonstrated a strong binding affinity to bovine serum albumin (BSA), suggesting the potential for effective drug delivery and bioavailability. Collectively, these findings highlight the potential of the investigated heteronuclear Au(I)-Cu(I) and Au(I)-Zn(II) complexes as promising anticancer agents, particularly against colon cancer, through their ability to disrupt redox homeostasis and induce oxidative stress-mediated cell death.

## 1. Introduction

GLOBOCAN 2020 reported over 19 million cancer cases and about 10 million cancer-related deaths globally [1]. Colorectal cancer (CRC) is ranked third in diagnoses and second in cancer-related deaths, which emphasises its impact. The use of platinum-based chemotherapic drugs is a common treatment option for CRC patients, particularly in advanced stages or when the cancer has spread to other parts of the body. These chemotherapic drugs are often administrated in combination with other agents, such as 5-fluorouracil, leucovorin, and capecitabine [2]. However, although platinum-based chemotherapy has shown effectiveness in shrinking tumours, controlling cancer growth, and improving survival rates in CRC patients, this treatment can also cause severe side effects [3].

To overcome such limitations, researchers are actively exploring potential alternatives to platinum-based chemotherapy using other metals [4,5], including gold. The exploration of gold complexes as metal-based drugs has been shown considerable attention, and a wide range of derivatives have been assessed for different biological activities, such as antibiotics [6], antifibrotic [7], and anticancer activity [8,9].

Besides mononuclear complexes, heterometallic derivatives have gained great attention and have been investigated for cancer therapy during the last few years. The incorporation of two different cytotoxic metals within a single molecule could enhance their efficacy as antitumor agents. This enhancement could be attributed to their interaction with multiple biological targets or to the improved physiochemical properties of the resulting hetero-polymetallic compound. Additionally, the presence of different mechanisms of action can lead to synergistic effects, modulation in redox properties, and changes in complex stability, which could result in an enhanced antitumor activity when compared to the mononuclear precursor [10,11,12].

The selection of the two metals that will form the heteronuclear complex is essential since it makes the therapeutic potential possible by synergistic effect or introduces significant features such as traceability, leading to theragnostic agents [11,13]. Since the serendipitous discovery of the anticancer activity of cisplatin, platinum-based complexes have undoubtedly become a milestone in inorganic medicinal chemistry. Regarding heterometallic complexes, they constitute the most widely studied family of derivatives [11]. Many examples of Pt-containing heterometallic compounds have been described in the last few years, showing great potential for overcoming the limitations of conventional Pt-based anticancer agents, including drug resistance, side effects, and diagnostic functions. Such examples include a combination of platinum with gold [14,15,16], ruthenium [17,18,19,20], titanium [21], iron [22,23], rhodium [24], copper [25], palladium [26], iridium [27], zinc [28], and luminescent metals [13,29,30], affording theragnostic heterometallic complexes.

Active heteronuclear gold complexes are less represented in comparison to platinum. A few examples of Au-containing heterometallic complexes Au-M (M = Pt, Ru, Fe, Ti, Cu, Ag) [14,21,31,32,33,34,35,36] have been reported, with a significant improvement in cytotoxic properties compared with the mononuclear precursor. Some of us have previously described Au(I)-Cu(I) complexes with enhanced anticancer activity attributable to a synergistic effect between the two metallic centres [35,36].

Some metal ions, such as Cu and Zn, are bioactive and essential elements for human life [37] and vital in many physiological processes [38]. Because of its importance, any malfunctions of homeostasis can lead to different disease states, also related to cancer development [39]. On the other hand, the coordination copper- or zinc-based compounds has demonstrated effectiveness in cancer therapy due to their cytotoxic action against various cancer cells [40,41]. Besides, these compounds are endogenously biocompatible and exhibit fewer adverse effects.

With this background, we describe here the preparation of heteronuclear complexes of gold with copper or zinc, by reacting the active gold complex [Au(Spy)(PTA)] (Spy = 2-thiolpyridine; PTA = 1,3,5-triaza-7-phosphaadamantane) [42], which acts as metalloligand with copper or zinc salts. Their anticancer activity has been evaluated against two human colon cancer cell lines. Additionally, we have also studied the potential anticancer mechanism of the most active complex, the Au_2_Cu derivative, including its antiproliferative effect, cell death effectors, and known targets of metallic compounds such as thioredoxin reductase.

## 2. Materials and Methods

General Procedures. All reactions were conducted under an argon atmosphere using standard Schlenk techniques. Proton (^1^H), phosphorous (^31^P), and carbon (^13^C) NMR spectra were recorded on a Bruker Avance 400 spectrometer, Billerica, MA, USA (400 MHz for ^1^H; 161.97 MHz for ^31^P; 100.62 MHz for ^13^C) using DMSO-d_6_ or CDCl_3_ as the solvents. Peak positions are relative to external tetramethylsilane (^1^H, ^13^C) or 85% H_3_PO_4_ (^31^P) and given in parts per million (ppm). Coupling constants are reported in Hz. MALDI mass spectra were measured on a Micromass Autospec spectrometer using DIT or DCTB as a matrix in positive ion mode. IR spectra were obtained on a Perkin Elmer Spectrum One FT-IR spectrometer (Waltham, MA, USA). Those samples in a solid state were measured using an ATR accessory covering a wavelength range from 4000 to 200 cm^−1^. UV-vis absorption spectra were recorded on a Thermo Scientific (Waltham, MA, USA) Evolution 600 spectrophotometer from solutions in quartz cuvettes (1 cm optical path). Elemental analyses were obtained in-house using a Perkin Elmer 2400 Series II CHNS/O System analyser. Fluorescence emission spectra were recorded on a Jobin-Yvon Horiba Fluorolog FL-3-11 spectrophotometer (Kyoto, Japan), using quartz cuvettes (1 cm optical path) for samples in solution.

Materials. The phosphane PTA, [AuCl(PTA)], and [Au(Spy)(PTA)] were synthesised as detailed in other references [43,44]. All chemicals were reagent-grade and were used as received by commercial suppliers.

### 2.1. Synthesis of Complexes

[Cu{Au(Spy)(PTA)}_2_]PF_6_ (**1**). To a solution of [Au(Spy)(PTA)] (0.2 mmol, 92.9 mg) in degassed and dried dichloromethane, [Cu(MeCN)_4_]PF_6_ (0.1 mmol, 37.3 mg) was added under an argon atmosphere. The clear yellow solution was stirred for 4 h at room temperature. A suspension gradually formed, and the solid was removed by filtration. The solvent was evaporated from the residue to a minimum volume, resulting in a yellow precipitate upon the addition of hexane. ^1^H NMR (400 MHz, CDCl_3_, 25 °C): δ = 8.20 (br s, 2H, HPy), 7.45 (d, J = 7.4 Hz, 1H, HPy), 7.37 (t, J = 7.4 Hz, 2H, HPy), 6.92 (br s, 2H, HPy), 4.56 and 4.47 (AB system, J_AB_ = 12.0 Hz, 12H, N + CH_2_N), 4.36 (s, 12H, N + CH_2_P) ppm. ^31^P{^1^H} NMR (162 MHz, CDCl_3_, 25 °C): δ = −52.4 (s, PTA), −144.0 (sept, J = 713.8 Hz, PF_6_^−^) ppm. ^13^C{^1^H} NMR (101 MHz, CDCl_3_, 25 °C): δ = 167.5 (s, 2C, C^2^), 148.7 (s, 2C, C^6^), 136.8 (s, 2C, C^4^), 128.0 (s, 2C, C^3^), 120.2 (s, 2C, C^5^), 73.4 (d, J = 7.5 Hz, 6C, NCH_2_N), 52.9 (d, J = 20.0 Hz, 6C, NCH_2_P) ppm. ^19^F{^1^H} NMR (376.52 MHz, CDCl_3_, 25 °C): δ = −72.1 (d, J = 711.2 Hz, PF_6_^−^) ppm. IR ν_max_/cm^−1^: 835, 556 (PF_6_^−^); 283 (Au-S). MALDI MS *m*/*z* (%): 465.04 (73) [Au(PTA)(Spy) + H]^+^, 511.11 (61) [Au(PTA)_2_]^+^, 526.98 (6) [CuAu(Spy)(PTA)]^+^, 677.02 (1) [M-2PTA]^+^, 818.12 (100) [Au_2_(Spy)(PTA)_2_]^+^, 833.99 (26) [M-PTA]^+^. Anal. calc. (%) for C_22_H_32_Au_2_CuF_6_N_8_P_3_S_2_ (1137.06): C, 23.24; H, 2.84; N, 9.85, S, 5.64. Found: C, 23.32; H, 2.81; N, 9.29; S, 5.57%. S 25 °C, H_2_O: <0.5 g/L.

[Zn{Au(PTA)(Spy)}_2_](NO_3_)_2_ (**2**). To a solution of [Au(PTA)(Spy)] (0.1 mmol, 46.4 mg) in methanol, Zn(NO_3_)_2_∙4H_2_O (0.05 mmol, 13.5 mg) was added. The clear solution was left stirring for 2 h at room temperature. After this time, the solvent was evaporated to a minimum volume and a white precipitate was obtained after the addition of diethyl ether. The solid was then filtered, washed with diethyl ether, and dried in air. Produced 66% yield, greenish-white solid. ^1^H NMR (400 MHz, DMSO-d_6_, 25 °C): δ = 8.13 (d, J = 4.1 Hz, 2H, H^6^), 7.36 (td, J = 7.9, 1.6 Hz, 2H, H^4^), 7.29 (d, J = 7.9 Hz, 2H, H^3^), 6.87 (ddd, J = 7.0, 5.0, 1.1 Hz, 2H, H^5^), 4.53 and 4.35 (AB system, J_AB_ = 12.0 Hz, 12H, NCH_2_N), 4.35 (s, 12H, NCH_2_P) ppm. ^31^P{^1^H} NMR (162 MHz, DMSO-d_6_, 25 °C): δ = −47.5 ppm. ^13^C{^1^H} NMR (101 MHz, DMSO-d_6_, 25 °C): δ = 167.5 (s, 2C, C^2^), 148.3 (s, 2C, C^6^), 135.5 (s, 2C, C^4^), 126.3 (s, 2C, C^3^), 117.9 (s, 2C, C^5^), 71.8 (d, J = 8.0 Hz, 6C, NCH_2_N), 51.0 (d, J = 19.7 Hz, 6C, NCH_2_P) ppm. IR ν_max_/cm^−1^: 1362, 1330, 1303 (NO_3_^−^), 288 (Au-S). MALDI MS *m*/*z* (%): 1117.94 (4) [M](NO_3_)_2_, 465.01 (66) [Au(PTA)(Spy) + H]^+^, 511.08 (16) [Au(PTA)_2_]^+^, 818.04 (100) [Au_2_(Spy)(PTA)_2_]^+^. Anal. calcd. (%) for C_22_H_32_Au_2_N_10_O_6_P_2_S_2_Zn (1117.94): C, 23.64; H, 2.89; N, 12.53, S, 5.74. Found: C, 23.19; H, 2.89; N, 12.15; S, 5.68%.

[Zn{Au(PTA)(Spy)}_2_]Cl_2_ (**3**). To a solution of [Au(PTA)(Spy)] (0.1 mmol, 46.4 mg) in methanol, ZnCl_2_ (0.05 mmol, 6.8 mg) was added. A white precipitate was formed immediately and the resulting suspension was left stirring for 1 h. After this time, the solid was filtered, washed with methanol and diethyl ether, and left to dry in air. Produced 96% yield, greenish-white solid. ^1^H NMR (400 MHz, DMSO-d_6_, 25 °C): δ = 8.13 (d, J = 4.2 Hz, 2H, H^6^), 7.36 (td, J = 7.7, 1.8 Hz, 2H, H^4^), 7.30 (d, J = 7.9 Hz, 2H, H^3^), 6.87 (ddd, J = 7.4, 4.9, 0.8 Hz, 2H, H^5^), 4.53 and 4.35 (AB system, J_AB_ = 12.0 Hz, 12H, NCH_2_N), 4.35 (s, 12H, NCH_2_P) ppm. ^31^P{^1^H} NMR (162 Hz, DMSO-d_6_, 25 °C): δ = −47.6 ppm. ^13^C{^1^H} NMR (101 MHz, DMSO-d_6_, 25 °C): δ = 167.5 (s, 2C, C^2^), 148.2 (s, 2C, C^6^), 135.5 (s, 2C, C^4^), 126.4 (s, 2C, C^3^), 117.8 (s, 2C, C^5^), 71.8 (d, J = 8.0 Hz, 6C, NCH_2_N), 51.0 (d, J = 19.6 Hz, 6C, NCH_2_P) ppm. IR ν_max_/cm^−1^: 292 (Au-S), 258 (Zn-Cl). MALDI MS *m*/*z* (%): 464.97 (1) [Au(PTA)(Spy) + H]^+^, 511.05 (3) [Au(PTA)_2_]^+^, 818.04 (100) [Au_2_(Spy)(PTA)_2_]^+^. Anal. calcd. (%) for C_22_H_32_Au_2_Cl_2_N_8_P_2_S_2_Zn (1064.84): C, 24.81; H, 3.03; N, 10.52, S, 6.02. Found: C, 24.05; H, 3.17; N, 10.63; S, 5.99%.

Distribution coefficient (logP_7.4_). The n-octanol-water coefficients of the complexes were determined using a shake-flask method, as previously reported [45]. Briefly, buffered-saline distilled water (100 mL, phosphate buffer [PO_4_^3−^] = 10 mM, [NaCl] = 0.15 M, pH 7.4) and n-octanol (100 mL) were shaken together for 72 h to allow for saturation of both phases. Approximately 1 mg of the complexes was dissolved in 5 mL of the aqueous phase and 5 mL of the organic phase was added, mixing them for 10 min. The resulting emulsion was centrifuged to separate the phases. The concentration of the compounds in each phase was determined using UV-vis absorbance spectroscopy. The logP_7.4_ was defined as log{[compound(organic)]/[compound(aqueous)]}.

Solution chemistry. The stability of the dinuclear derivatives was analysed by absorption UV-vis spectroscopy. UV-vis absorption spectra of the complexes were recorded on a Thermo Scientific spectrophotometer. Solutions of the complexes (5 × 10^−5^ M) in PBS (pH = 7.4) were prepared from 20 mM DMSO stock solutions and thereafter monitored over 24 h at 37 °C, measuring the electronic spectra.

Interaction with bovine serum albumin. BSA was purchased from Sigma Aldrich (St. Louis, MO, USA). A 2 mM stock solution of BSA was prepared by dissolving the commercial powder in PBS at pH 7.4. Afterwards, the real concentration was confirmed using UV-vis spectroscopy (ε_280_ nm = 43,824 M^−1^cm^−1^). The heteronuclear complexes were dissolved in DMSO to achieve 6 mM stock solutions, and six aliquots of 2.5 μL were added to a 50 μM solution of BSA in PBS placed in a quartz cuvette of 1 cm optical path. The final concentrations of the complexes in the cuvette were 0, 5, 10, 15, 20, 25, and 30 μM. The fluorescence spectra were recorded on a Jobin-Yvon-Horiba fluorolog FL-3-11 spectrometer. The samples were excited at 295 nm and the emission spectra were recorded in a range from 310 to 450 nm.

The samples were measured 4 min after every addition of the aliquots of the complexes. The fluorescence intensities of the PBS and the complexes were irrelevant under the described conditions. The data were analysed using the Stern–Volmer equation F_0_/F = 1 + K_SV_ [gold complex] = 1 + K_q_τ0[complex] to obtain the Stern–Volmer quenching constant (K_SV_) and the quenching rate constant (K_q_). The binding constant (K_b_) was quantified by using the Stern–Volmer equation: log {(F_0_ − F)/F} = log K_b_ + n log [complex].

Cell culture. Human colon cancer Caco-2 cells (PD7 and TC7 clones) [46,47] were kindly provided by Dr. Edith Brot-Laroche (Université Pierre et Marie Curie-Paris 6, UMR S 872, Les Cordeliers, Lyon, France) and grown in Dulbecco’s Modified Eagles medium (DMEM) (Gibco Invitrogen, Paisley, UK) supplemented with 20% foetal bovine serum (FBS), 1% non-essential amino acids, 1% streptomycin (1000 μg/mL), 1% penicillin (1000 μg/mL), and 1% amphotericin (250 U/mL). Caco-2 cells were maintained at 37 °C in a humidified atmosphere at 5% CO_2_.

For cell viability studies of differentiated (enterocyte-like) cells, Caco-2/PD7 cells were seeded on 96-well plates at a density of 4 × 10^3^ cells/well and incubated for approximately 10–15 days, changing the culture medium every 3 days, up to reaching confluence—the moment in which the cells were treated with the complexes at concentrations equal to their mean IC_50_ and 2 × IC_50_ values.

Cell proliferation assay. Cell proliferation was measured using the 3-(4,5-dimethyl-2-thiazoyl)-2,5-diphenyltetrazolium bromide (MTT) assay, as previously described [48]. Caco-2/TC7 cells were seeded in a 96-well plate at a density of 4000 cells/well and grown for 24 h before being treated with each complex or DMSO (used as control). After 72 h of incubation with the complexes, 10 μL of MTT (5 mg/mL) were added and incubation was continued at 37 °C for 3 h. Inversion removed the medium and 100 μL of DMSO were added to each well. Absorbance at 560 nm, proportional to many living cells, was measured by spectrophotometry (SPECTROstar Nano, BMG LABTECH, Ortenberg, Germany) and converted into a percentage (%) of growth inhibition.

Study of cell death. After the appropriate incubation, cells were collected and stained with annexin V-FTIC, according to the manufacturer’s instructions. A negative control was prepared, containing unreacted cells to define the basal level of apoptotic and necrotic or dead cells. After incubation, the cells were transferred to flow-cytometry tubes and washed twice with temperate phosphate-buffered saline (PBS), suspended in 100 μL annexin V binding buffer (10 mM Hepes/NaOH, pH 7.4, 140 mM NaCl, 2.5 mM CaCl_2_), then 5 μL of annexin V-FITC, and 5 μL of PI was added to each 100 μL of cell suspension. After incubation for 15 min at room temperature in the dark, 400 μL of 1× annexin binding buffer was added and analysed by flow cytometry within one hour. The signal intensity was measured using a FACSARIA BD and analysed using FASCDIVA BD.

Cell cycle analysis. After treatment, the cells were fixed in 70% ice-cold ethanol and stored at 4 °C for 24 h. After centrifugation, the cells were rehydrated in PBS (phosphate-buffered saline) and stained with propidium iodide (PI, 50 μg/mL) solution containing RNase A (100 μg/mL). PI-stained cells were analysed for DNA content in a FACSARRAY BD equipped with an argon ion laser. The red fluorescence emitted by PI was collected using a 620 nm longer pass filter as a measure of the amount of DNA-bound PI and displayed on a linear scale. Cell cycle distribution was determined on a linear scale. The percentage of cells in cycle phases was determined using MODIFIT 3.0 verity software.

Measurement of intracellular ROS generation. The production of ROS was measured in Caco-2/TC7 seeded at a 4000 cells/well concentration in a 96-well plate, as described above. After 24 h, the culture growth medium was removed and cells were washed with PBS 1× and incubated with 20 µM DCFH-DA (Sigma-Aldrich) (100 µL/well) at 37 °C for 45 min in the dark. Afterwards, cells were washed with PBS 1× and then incubated with the tested compounds at their IC_50_ concentration or with 500 µM of H_2_O_2_ for the positive control. The fluorescent emission of DCF was monitored at 530 nm right after the addition of the complexes for 3 h using a microplate reader (λ_ex_ = 485 nm), measuring every 15 min. The temperature was maintained at 37 °C during the experiment.

Determination of the activity of thioredoxin reductase (TrxR). The method is based on the reaction of 2,2′-dinitro-5,5′-dithiobenzoic acid (DTNB) with thiol groups of proteins to form the 2-nitro-5-thiobenzoate anion (TNB^2−^), which absorbs at λ_max_ = 412 nm. The Caco-2 cell line (TC7 clone) was seeded in a 25 cm^2^ flask (2 × 10^6^). After 24 h of incubation, the IC_50_ concentration of compound **2** was added for 24 h. Then, the cells were collected and lysed by M-PER^®^ Mammalian Protein Extraction Reagent (reference commercial Thermo Fisher Scientific, 78501), following the supplier’s instructions. The interaction of metal complexes with the enzyme thioredoxin reductase was analysed using a thioredoxin reductase assay kit (Sigma, CS0170, St Louis, MO, USA). The procedural guidelines of the supplied kit were followed. The reaction started with the introduction of DTNB (100 mM), and the transformation into TNB was observed at 412 nm at 30 s intervals over 5 min, utilising a SPECTROstar Nano multi-plate reader from BMG Labtech. The outcomes were presented as a percentage representing the TrxR activity relative to the control. The non-treated cells’ activity was considered the control and estimated as 100% of activity.

### 2.2. DNA Binding Constant Calculation

A ctDNA solution was prepared at 1 mg/mL in tris (tris(hydroxymethyl)aminomethane)/HCl (0.1 M, pH 7.2). Then, the purity of the ctDNA was checked, recording its spectra and calculating the absorption ratio between 260 and 280 nm. This experiment also provides the means to calculate the concentration of ctDNA using the Beer–Lambert formula, with the extinction coefficient of 6600 M^−1^cm^−1^. Every experiment was corrected with a baseline of the buffer and the corresponding concentration of dimethylsulfoxide. The addition of DNA solution was performed in both assay and reference cuvettes. Then, the binding constant was calculated using the modified Benesi–Hildebrand equation. K_b_ is the binding constant, ε_f,_ ε_a,_ and ε_b_ are the extinction coefficients for the free compound, the apparent coefficient (calculated as the ratio of the absorbance and the concentration of the compound in the experiment), and the compound–DNA complex, respectively. Plotting [DNA]/(ε_f_ − ε_a_) vs. [DNA] (Figures S18 and S20) and performing a lineal fitting gives as a result a line equation y = mx + n, where m/n gives the binding constant.

$$\frac{\left[DNA\right]}{{\varepsilon_{f}-\varepsilon }_{a}}=\frac{\left[DNA\right]}{{\varepsilon_{f}-\varepsilon }_{b}}+\frac{1}{{{k_{b}(\varepsilon }_{f}-\varepsilon }_{b})}$$

### 2.3. DNA Interaction

Adducts with pIRES2-EGFP (5308 pb) plasmid DNA (Catalog #6029-1) were prepared by adding the required volume of a freshly prepared solution of metal complexes in MilliQ water. The concentration of pIRES2-EGFP in the reaction mixture was 117 ng/µL, and the complexes’ concentration was varied to give different metal-to-base pair stoichiometries (0.5, 1 and 2). The mobility of the metal complex-treated pIRES2-EGFP samples was analysed by gel electrophoresis on a 0.8% (*w*/*v*) agarose gel (Boehringer-Mannheim, Mannheim, Germany) at 90 V/cm at 25 °C in Tris-acetate/EDTA buffer. The gel was stained for 30 min in 0.5 g/mL (*w*/*v*) ethidium bromide and the bands were visualised with a UVP gel scanner.

## 3. Results and Discussion

### 3.1. Synthesis and Characterisation

**Scheme 1:**
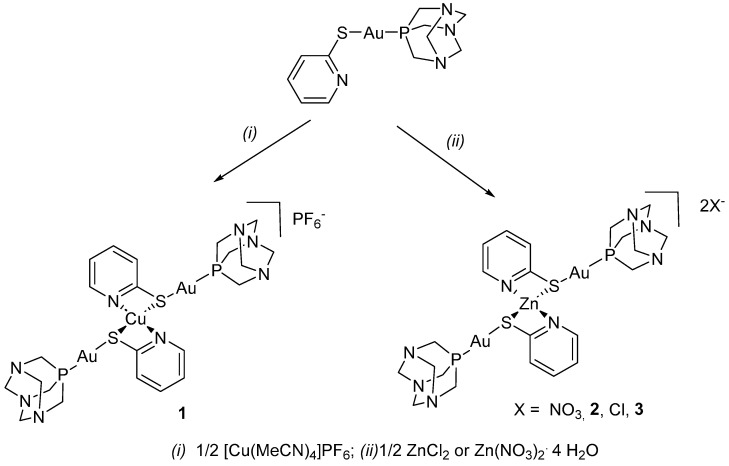
Synthesis of the heterometallic complexes.

The reaction of [Au(Spy)(PTA)] (PTA = 1,3,5-triaza-7-phosphaadamantane) [49] with tetrakis(acetonitrile)copper(I) hexafluorophosphate in a 2:1 ratio under argon atmosphere led to a yellow solid that analyses as a trinuclear derivative [Cu{Au(Spy)(PTA)}_2_](PF_6_) (**1**). The use of Zn(NO_3_)_2_ or ZnCl_2_ in a 2:1 ratio in methanol affords the complexes [Zn{Au(Spy)(PTA)}_2_](NO_3_)_2_ (**2**) and [Zn{Au(Spy)(PTA)}_2_]Cl_2_ (**3**) as white, analytically pure solids in fair yield (Scheme 1).

The coordination of copper(I) to two units of the metalloligand (complex **1**) is revealed in the ^1^H NMR spectrum by the shift to a lower field of the thiolpyridine proton signals. In contrast, the protons of the PTA moiety remain very similar regarding the mononuclear precursor. In the ^31^P{^1^H} NMR spectra, a singlet can be observed at −50.8 ppm in DMSO-d_6_ and −52.4 ppm in CDCl_3_, which implies a slight upfield shift regarding the signals of the mononuclear complexes (−48.5 ppm in DMSO-d_6_ and −50.3 ppm in CDCl_3_). The characteristic septuplet of the PF_6_− counterion can also be observed at −144 ppm. These changes are consistent with what has been previously observed for the heteronuclear complexes [Cu{Au(C≡CCH_2_SC_5_H_4_N)(PR_3_)}_2_]PF_6_ (PR_3_ = PPh_3_, PTA) described by some of us [35].

The pattern of signals of both Au_2_Zn complexes, **2** and **3**, in the ^1^H NMR spectra (Table 1) remains very similar to that observed for their mononuclear precursor, with the only difference arising from the signals of protons in positions 3 and 4 in the pyridine ring, which appear as a multiplet in the precursor [Au(Spy)(PTA)], as a resolved doublet at 7.29, and a triplet of doublets at 7.36 ppm, respectively. The signal of the phosphorus atom in the ^31^P{^1^H} NMR spectrum appears as a singlet at around −47 ppm, which implies a very slight downfield shift regarding the signal of the free metalloligand (complex **1**), in agreement with what has been observed in other Au_2_Zn heteronuclear complexes [50]. With only this information available, it is not possible to assign the coordination environment of the zinc atom, which could be either by the N and S atoms of the pyridine group or by N-coordination to the PTA moieties. Smoleński et al. [51] described the first Zn-PTA complex, [ZnCl_2_(PTA)_2_], in which the metallic centre is coordinated to two units of PTA by one of the nitrogen atoms of the moiety. N-coordination is justified by the changes in the ^1^H NMR spectrum: a doublet for the methylene protons of NCH_2_P and a singlet for the methylene protons of NCH_2_N, in contrast with the broad singlet and AB system, respectively, of P-coordinated PTA. The free metalloligand is already P-coordinated to a gold(I) atom, so the changes would be expected for the methylene protons of the NCH_2_N groups. But these protons remain very similar in both complexes **2** and **3** regarding the signals of the free metalloligand [Au(Spy)(PTA)]. To acquire further information on the coordination mode of the zinc(II) atom, ^1^H NMR spectra at different temperatures of **2** in MeOD were recorded (Figure S13, Supplementary Materials). Unfortunately, the poorer solubility of the heteronuclear complex in MeOD, while decreasing the temperature, prevented us from concluding the coordination mode using NMR.

Their IR spectra display strong bands between 1540 and 1650 cm^−1^ due to the υ(C=C) and υ(C=N) of the pyridine ring [52]. These aromatic bands remain very similar for the heteronuclear complexes and their mononuclear precursor, except for the appearance of a new band at ~ 1590 cm^−1^ for the Au_2_Cu and Au_2_Zn complexes **1**, **2,** and **3** (see Table 2). Bands associated with ring breathing vibration are located between 1150 and 990 cm^−1^, and 780 and 620 cm^−1^. The strong band at 1159 cm^−1^ in the IR spectrum of [Au(Spy)(PTA)] becomes very weak in the spectra of the heteronuclear complexes, and a new band appears in this region (1138 cm^−1^) for the Au_2_Zn compounds **2** and **3**. It is worth noting the shift undergone by the bands of the free metalloligand at 783 cm^−1^ and 625 cm^−1^, caused by ring deformation vibrations, which appear at ca. 757 cm^−1^ and 648 cm^−1^, along with a decrease in their intensity in the spectra of all the heteronuclear complexes. The shift in these two bands is indicative of metal coordination to the thiolpyridine moiety, specifically through the nitrogen atom [52,53,54]. Bands due to the vibrations of the PTA group remain very similar for all the mono- and heteronuclear complexes. In the far region, some other bands are present due to the υ(Au-S) and υ(Au-P) at 289 cm^−1^ and 270 cm^−1^, and they do not differ much for all the spectra.

### 3.2. Solution Stability

The solution stability of the new heterometallic compounds was determined under physiological conditions. To simulate conditions similar to those in cell culture and the body, the compounds were dissolved in a phosphate-buffered saline solution (PBS) with a pH of 7.4 and then incubated at 37 °C. The UV spectra of each complex were recorded at different time intervals (Figure S14). Qualitative determination of the stability of the compounds under such conditions was achieved by comparing the spectra obtained at each time point. The spectrum of the Au_2_Zn complex, [Zn{Au(PTA)(Spy)}_2_](NO_3_)_2_ (**2**), remained unaltered for a period of 24 h, indicating that it is highly soluble and stable under physiological conditions. However, the Au_2_Cu complex, [Cu{Au(Spy)(PTA)}_2_]PF_6_ (**1**), displays reduced solubility in PBS, resulting in the formation of a suspension during the incubation period. Nevertheless, no discernible red- or blueshift is observed after incubation in both complexes, and neither with absorbance at around 500 nm—which would be indicative of gold nanoparticle formation—over 24 h, suggesting their notable stability under physiological conditions.

### 3.3. BSA Interaction

Plasma protein binding is a crucial step for drugs once they enter the bloodstream, influencing their distribution, absorption, and elimination from the body. This process impacts the drug’s pharmacokinetic and pharmacodynamic profile, making the study of plasma protein binding essential when determining the ADMET properties of new compounds. Due to their similarity, bovine serum albumin (BSA) is often used as a model protein for human serum albumin (HSA), the most abundant protein in plasma. Therefore, the study of the interaction of gold drugs with BSA can provide information on the distribution, free concentration, metabolism, and efficacy of the gold compound [55].

**Figure 1:**
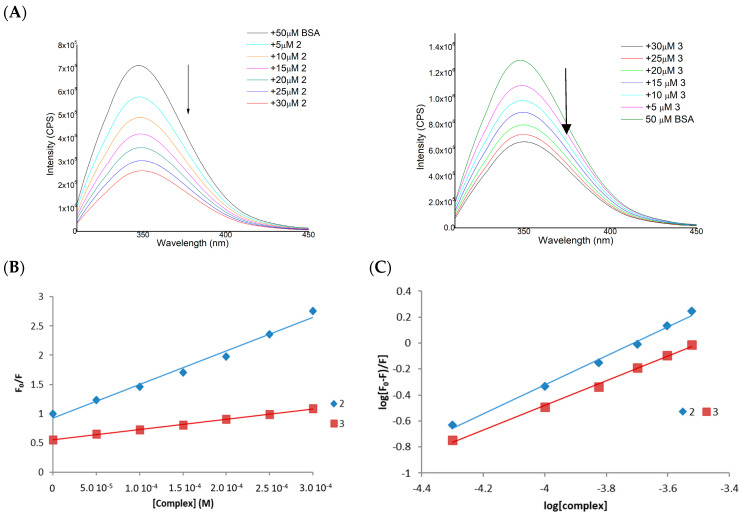
(**A**) Fluorescence emission spectra of BSA in the presence of different concentrations of [Cu{Au(Spy)(PTA)}_2_]PF_6_ (**1**) (**A left**) and [Zn{Au(PTA)(Spy)}_2_](NO_3_)_2_ (**2**). (**A right**) (**B**) Stern−Volmer plots for the quenching of BSA with increasing amounts of complexes **1** and **2** (complex concentration from 0 to 30 μM) at 298 K (λ_exc_ = 295 nm, [BSA] = 50 μM). Stern−Volmer equation used: F_0_/F = 1 + K_SV_[complex]. The slope of the best fit linear trend provides the Stern−Volmer quenching constant K_SV_. (**C**) Stern−Volmer equation used: log{(F_0_ − F)/F} = log K_b_ + n log[complex]. The intercept of the best fit linear trend provides the Stern−Volmer quenching constant K_b_.

We have carried out fluorescence and UV-Vis spectroscopic experiments to study the affinity of [Cu{Au(Spy)(PTA)}_2_]PF_6_ (**1**) and [Zn{Au(PTA)(Spy)}_2_](NO_3_)_2_ (**2**) for albumin. These experiments are based on the quenching of the intrinsic tryptophan fluorescence emission undergone by BSA in the presence of increasing concentrations of the trinuclear complexes, which act as quenchers. The intrinsic fluorescence of BSA is susceptible to alterations in its local environment, and the binding or simple electrostatic interaction of molecules can result in modifications to its emission spectrum [56]. Fluorescence quenching experiments have been carried out, observing the emission of tryptophan at 341 nm upon excitation at 295 nm and increasing concentrations of compounds **2** and **3** with BSA. Before performing the quenching analysis, the fluorescence of the tested complexes was measured under the same experimental conditions, and no emission was observed in the range 295–300 nm. The fluorescence emission spectra of BSA after the interaction with trinuclear complexes are shown in Figure 1A. The addition of increasing amounts of the complexes to a BSA solution results in a significant reduction in fluorescence intensity without any shift in the emission maximum. The fluorescence data have been analysed using the Stern–Volmer equation so that the binding of the complexes to BSA and the consequent quenching of the emission can be quantified (Table 3). The corresponding plot F_0_/F versus [complex] (F and F_0_ are the corresponding intensities in the presence and the absence of the quencher agent) gives a linear plot in both cases (Figure 1B), characteristic of the presence of a single mechanism of quenching, either static or dynamic [57]. Static quenching implies the formation of a complex involving the quencher and the protein, generating deviations from linearity in Stern–Volmer plots, although only observable at high quencher concentrations. Collisions between the protein and the quencher cause dynamic quenching.

The quenching constants values (K_SV_)—around 10^3^ M^−1^—and the binding constants (K_b_) (Figure 1C)—in the range 10^3^–10^4^ M^−1^—remain in the same order of magnitude as previously described for mononuclear gold complexes [58,59,60] and mononuclear copper or zinc derivatives [61,62]. This fact indicates that the trinuclear complexes exhibit a high affinity towards BSA, with [Cu{Au(Spy)(PTA)}_2_]PF_6_ (**1**) showing the highest affinity.

**Figure 2:**
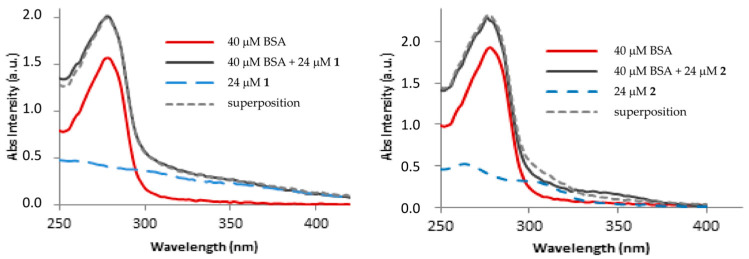
Absorption spectra of BSA (red solid line), compounds [Cu{Au(Spy)(PTA)}_2_]PF_6_ (**1**) and [Zn{Au(PTA)(Spy)}_2_](NO_3_)_2_ (**2**) (dashed blue line), BSA + compound (black solid line), and their superposition (dotted grey line).

Furthermore, examination of the absorption spectra of the fluorophores can be used as a means of characterising static and dynamic quenching. Given the premise that static quenching entails complex formation, alterations in the absorption spectra of BSA are to be anticipated. Conversely, since dynamic quenching only affects the excited states of BSA, its absorption spectra would remain unaltered. The absorption spectra of BSA were recorded in the absence and presence of each compound at a concentration of 24 μM, as well as the absorption spectrum of the compound at the same concentration. A mathematical superposition of the spectrum of BSA and the spectrum of the compounds was calculated. In the case of complex **1**, the addition of both spectra can be superimposed on the spectrum of the BSA + complex, which implies dynamic binding. However, in the case of complex **2**, there is a slight deviation in the superposition, which suggests that the static binding mode may be implied (Figure 2).

### 3.4. Biological Studies

The antiproliferative effects of the mononuclear and heteropolinuclear complexes were tested on two different Caco-2 cancer cell lines (PD7 and PC7). Both clones exhibit elevated rates of glucose consumption and comparable functionalities, with the difference lying in their origins: PD7, correspondingly, was preceded by early passages, being more heterogeneous; on the other hand, TC7 showed a higher level of stability and homogeneity, as it stemmed from late passages. Caco-2 cells of both PD7 and TC7 clones were exposed to increasing concentrations of the assayed compounds through dose–response/cell viability analysis. The results in terms of the IC_50_ parameter (Table 4) were obtained by using the MTT protocol [63].

The cytotoxic activity of complex [Au(Spy)(PTA)], used as a metalloligand in this work, was already screened against ovarian cancer cells lines, showing IC_50_ values of 9.6 μM against A2780/S and 8.2 μM against the cisplatin-resistant A2780/R [42]. Its cytotoxic properties have improved against the colon cancer cell lines Caco-2, lowering the IC_50_ concentration to 2.64 and 2.03 μM in PD7 and TC7, respectively, which are values similar to those obtained for other gold(I) thiolate complexes [60,64] and in the same order as the reference drug auranofin. Introducing other metal ions to obtain new heteronuclear compounds has different effects on their cytotoxic activities, with a clear dependence on the nature of the new metallic centre. Thus, while the IC_50_ values obtained for the Au_2_Zn complex, [Zn{Au(Spy)(PTA)}_2_](NO_3_)_2_ (**2**), only show a slight improvement compared to the mononuclear precursor, the cytotoxic activity of the Au_2_Cu compound (**2**) greatly improves with IC_50_ concentrations 15-times lower compared to those of [Au(Spy)(PTA)]. It seems that the coordination of the Zn(II) ion would not play a decisive role in the cytotoxic activity of compound **2**. In contrast, the Cu(I) ion would play a relevant role in enhancing the biological anticancer activities. This improved activity could be attributed to better cellular uptake, derived from a higher lipophilic character of complex **1** compared to compound **2** (Table 4).

It is well known that the Caco-2 cell line undergoes spontaneous differentiation into enterocytic-like cells when it reaches confluency. As a result, these cells become polarised, expressing apical and basolateral surfaces with established tight junctions [65]. This allowed for the evaluation of the cytotoxic impact of the complexes on differentiated cells (acting as noncarcinogenic) and a comparison with the viability of undifferentiated cells. Thus, the toxicity of the two heteronuclear compounds has been studied by cell viability experiments by treatment of Caco-2/PD7 cells after differentiation with complexes **1** and **2** at the corresponding concentrations equivalent to IC_50_ and 2 × IC_50_ values for 72 h. The results expressed as the % of cell viability observed after treatment with the complexes (or 100% for non-treated control cells) are summarised in Figure S16 (Supplementary Materials). As observed in the graph, the most active complex against cancer cells [Cu{Au(Spy)(PTA)}_2_]PF_6_ (**1**) also turns out to be the most active against non-tumour cells. However, the cell viability is around 90% even when the cells are treated with double the IC_50_ concentration.

### 3.5. Apoptosis and Cell Cycle Studies

**Figure 3:**
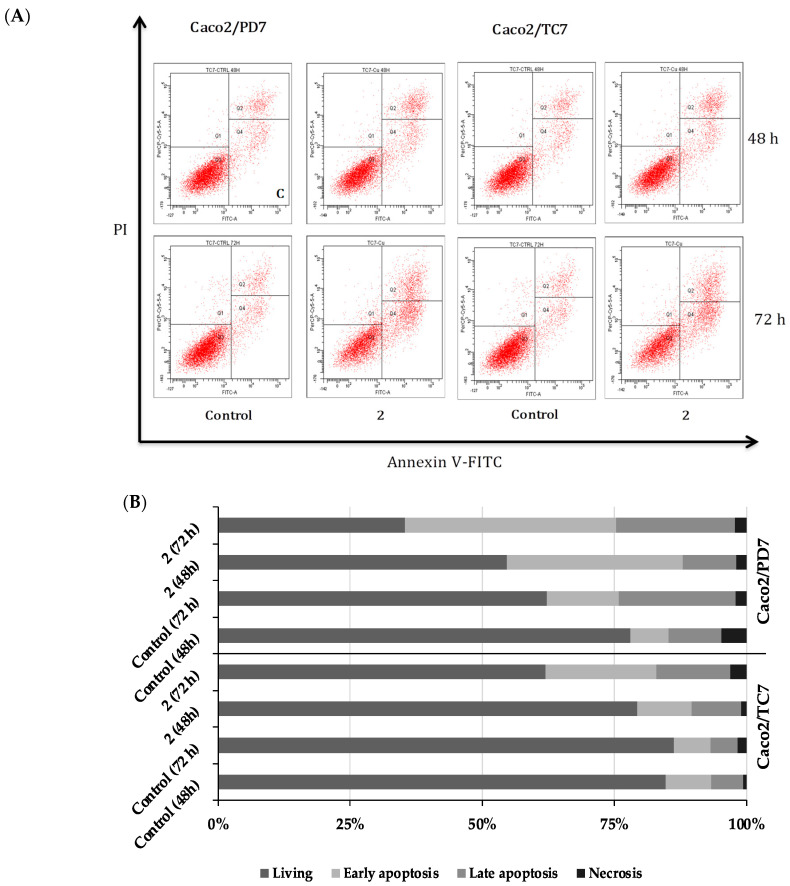
(**A**) Density plots obtained from analysis of Caco-2/TC7 and Caco-2/PD7 cells. Control: non-treated cells; [Cu{Au(Spy)(PTA)}_2_]PF_6_ (**1**); Caco-2 cells were stained with annexin V-FITC and PI after 48 and 72 h of incubation with the IC_50_ concentrations of **1**. (**B**) Cell death data.

Since the complexes can reduce cell viability, the annexin V/propidium iodide double-staining assay was carried out to quantify the most active complex-induced apoptosis by flow cytometry. Treatment of Caco-2/PD7 cells with the benchmark drug auranofin led to most cells being found in late-stage apoptosis and necrosis (Figure S17, Supplementary Materials). After 48 and 72 h of incubation with the most active derivative [Cu{Au(Spy)(PTA)}_2_]PF_6_ (**2**), a decrease in cell viability was observed (Figure 3A), as the proportion of living cells was reduced over time, with the consequent increase in cells undergoing apoptosis: from a 35.8% of apoptotic cells (early + late apoptosis) in the case of control cells to 62.4% of total apoptosis for cells incubated for 72 h. In the case of the TC7 clone, control cells displayed inhibition of the apoptotic process, with 85% of cells exhibiting viability and a relatively low proportion undergoing cell death. This inhibition of apoptosis produces a fast growth and proliferation of cancerous cells. The population of early and late apoptotic cells increased to 35% after treatment with complex **1** in comparison with the vehicle-treated cells (12%). These results point to the apoptosis-inducing ability of the heteronuclear derivative in Caco-2 cells. Additionally, the effect observed after treating the cells with a dosage equal to the IC_50_ concentration is time-dependent. Thus, the proportion of cells undergoing apoptosis increases throughout the time, with a maximal percentage of apoptotic cells found after 72 h of incubation/treatment and the proportion of necrotic cells remaining considerably low for all the experiments performed.

**Figure 4:**
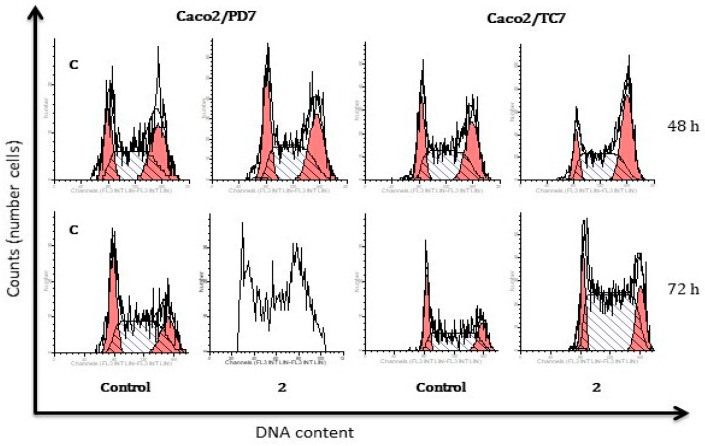
Effect of complex **1** on the cell cycle distribution of Caco-2/TC7 and Caco-2/PD7 cells after treatment with its IC_50_ concentrations for 48 h and 72 h.

To investigate the influence of the heteronuclear derivative **1** on the cell cycle distribution of Caco-2 cells, propidium iodide-stained cells were analysed by flow cytometry. The distribution of cells found in each stage of the cell cycle for the control samples was sufficiently homogeneous (Figure 4). For both cell lines, samples analysed after 48 h of incubation had 22% of the cells in the G0/G1 phase, ca. 45% of cells in the synthesis phase, and approximately 30% of cells undergoing the G2 phase or cell division. The low percentage of cells observed in the G0 and G1 phases may be indicative of elevated proliferation and an uncontrolled growth of cancerous cells. It would be of interest to ascertain whether the complexes chosen for the treatments could arrest the cell cycle at phases preceding cell division, thereby inhibiting the proliferation of cancer cells. The treatment with [Cu{Au(Spy)(PTA)}_2_]PF_6_ (**1**) appears to exert a significant influence on the cell cycle of Caco-2/PD7 cells, as evidenced by the observation of a comparable population after 48 h of incubation and not enough living cells being found for the analysis after 72 h. However, complex **1** has been shown to induce an arrest of the cell cycle in Caco-2/TC7 cells within the G0/G1 phases, accompanied by a notable reduction in the population within the S phase (Figure 4).

### 3.6. Effect of Heteronuclear Complexes on Intracellular Redox State

It has been proposed by some authors that manipulation of ROS (reactive oxygen species) levels may represent an effective strategy for the treatment of cancer [66,67]. This is on the grounds that cancerous cells are especially redox-vulnerable, and this could be used for treatment with antioxidant-lowering or ROS-producing drugs. An overproduction of ROS leads to oxidative stress, which induces damage to all the cell components: lipid peroxidation and consequent damage to lipid membranes and DNA damage [68].

It is important to study the effect caused by ROS production upon the treatment of cells with metallic complexes, as it could indicate TrxR (thioredoxin reductase) inhibition and induction of apoptosis. Thus, the production of hydrogen peroxide in Caco-2 cells upon treatment with IC_50_ concentrations of complexes **1** and **2** has been studied. The method based on the H_2_O_2_-sensitive fluorescent probe DCFH-DA (2′-7′dichlorofluorescin diacetate) has been used.

**Figure 5:**
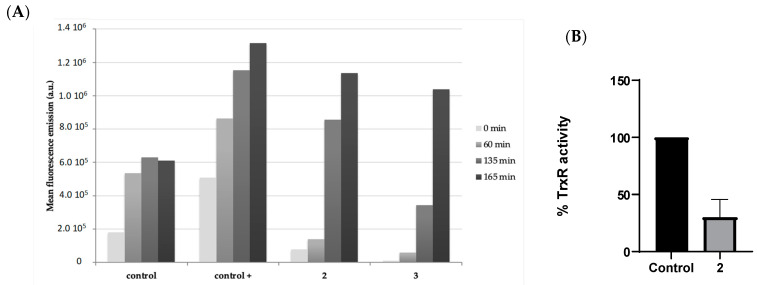
(**A**) Effect of heteronuclear complexes on H_2_O_2_ formation in Caco-2/PD7 cells. (**B**) Inhibitory effect of [Cu{Au(Spy)(PTA)}_2_]PF_6_ (**2**) on thioredoxin reductase (TrxR) at IC_50_ concentrations. TrxR activity was measured as the increase in absorbance at 412 nm per minute. Values represent the percentage of activity related to control.

Once cells were incubated with DCFH-DA, the two heteronuclear complexes were added, followed by the monitoring of the hydrogen peroxide basal production for 3 h, measuring the emission intensity of DCF (2′,7′-Dichlorofluorescein) every 15 min. H_2_O_2_ was added to the positive control cells. In the plots depicted in Figure 5A, an increase in the fluorescent emission of DCF can be observed, which is under an increase in basal H_2_O_2_ production. It is worth noting that there is low emission within the first 60 min of the experiment in both cases. This fact could be explained by the complexes not entering the cells within the first minutes of the experiment; however, after 60 min, there is a rise in the emission intensity, indicating ROS formation. As can be seen in Figure 5A, both compounds cause an increase in DCF fluorescence signals as compared with the vehicle-treated cells, which occurs more markedly in the case of [Cu{Au(Spy)(PTA)}_2_]PF_6_ (**1**).

### 3.7. Interaction with Thioredoxin Reductase

Thioredoxin reductase (TrxR) is an enzyme that plays a crucial role in maintaining cellular redox balance and protecting cells from oxidative stress [69]; consequently, the inhibition of TrxR has an impact on the production of oxidative species [70]. The overexpression of both cytoplasmic and mitochondrial TrxR isoforms (TrxR1, TrxR2) has been found in various types of cancer, including colon, breast, lung, oral cavity, and squamous cell carcinoma [71,72]. In the context of colon cancer, TrxR has been implicated in promoting cancer cell survival and proliferation by regulating redox signalling pathways. Furthermore, the expression of TrxR has been linked to drug resistance, cancer metastasis, and cancer survival [73]. Therefore, targeting TrxR in colon cancer cells could be a potential therapeutic strategy to disrupt redox homeostasis and induce cell death [74,75].

Prior research on gold derivatives demonstrated disruptions in reactive oxygen species balance when cancer cells were exposed to metal complexes [60,76,77]. This often results in elevated intracellular levels of ROS because certain gold(I) derivatives interact with thioredoxin reductase (TrxR), leading to the inhibition of its antioxidant function [9,60,76,78,79].

Therefore, we investigated the influence of the most active derivative, [Cu{Au(Spy)(PTA)}_2_]PF_6_ (**2**), on the activity of TrxR from undifferentiated Caco-2/TC7 cells after 24 h of incubation with it. As shown in Figure 5B, the heteronuclear complex inhibited TrxR activity after the 24 h incubation. In light of these findings, it can be posited that these heteronuclear complexes could interact with TrxR, thereby inhibiting its antioxidant activity and consequently leading to an increase in intracellular ROS levels.

### 3.8. DNA Binding

Given that copper has shown a rather high affinity for DNA, we have determined the possible interaction of the trinuclear complex Au_2_Cu (**1**) with ct-DNA (calf thymus DNA) by UV-vis and compared with the precursor [Au(Spy)(PTA)] (**1**). DNA offers different binding modes (outer-sphere, non-covalent binding, metal coordination to nucleobases, and phosphate backbone interaction) for metallic-based anticancer drugs [80,81]. DNA binding is frequently determined by UV-vis by recording the changes in the absorbance and shifts in wavelength. Drug–DNA interactions can be studied by comparing the UV-vis absorption spectra of the complex before and after the addition of DNA. The different spectral absorbances of DNA with complexes indicate the presence of interactions [82]. Complexes binding to DNA through intercalation afford hypochromism and batochromism (red shift). However, the hyperchromic effect is observed in the case of electrostatic interactions between the complex and DNA, which indicates changes in DNA conformation and structure after complex interaction [83].

**Figure 6:**
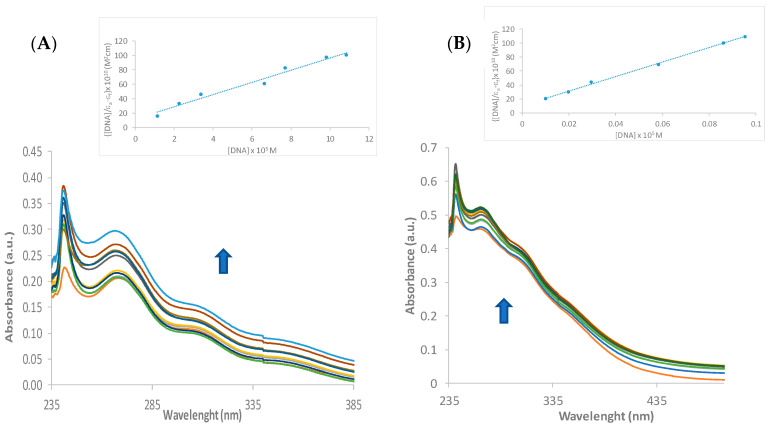
Electronic absorption spectra of complexes [Au(Spy)(PTA)] (**A**) and **1** **(B**) (20 μM) in Tris-HCl buffer (pH = 7.20) in the absence and the presence of increasing amounts of ct-DNA ([DNA] from 0 to 100 μM). Arrows show the changes in absorbance with increasing DNA concentration. Inset: plot of [DNA]/[εa − εf] vs. [DNA].

The binding capabilities of [Cu{Au(Spy)(PTA)}_2_]PF_6_ (**1**) and [Au(Spy)(PTA)] were estimated towards wheat DNA by recording the electronic absorption spectra throughout their interaction. The absorbance measurements were performed by successive additions of a DNA stock solution (10, 20, 30, 40 up to 100 μL) which afforded the final concentration of ct-DNA (from 0 to 100 μM) while maintaining the concentration of the complexes (20 μM). The electronic absorption study of both complexes showed an increase in the intensity of the bands centred around 240 and 260 nm (Figure 6). These spectral changes point to non-covalent interactions between the complexes and DNA, or simply to the uncoiled DNA double helix, and exposed more DNA bases. The non-covalent interactions may include hydrogen bonding and van der Waal’s attraction forces between the base pairs and the complexes. Hyperchromic shifts revealed the changes in DNA structure and conformation that occurred after the compounds bound to DNA, leading to structural damage to the DNA helix [82,83].

To quantify the interaction with DNA, we used the modified Benesi–Hildebrand equation (see experimental section). We determined values of 9.63 × 10^6^ (**1**) and 9.06 × 10^4^ ([Au(Spy)(PTA)]) M^−1^ for the binding constants (K_b_). The value of complex [Au(Spy)(PTA)] is consistent with the values reported for other gold derivatives [84,85]; however, the value calculated for the heteronuclear complex **1** is surprisingly higher and even higher than the classical intercalator EB (ethidium bromide) binding affinity for ct-DNA, (K_b_ = 1.23(±0.07) × 10^5^ M^−1^) [86], which suggests that this complex has a strong binding affinity for ct-DNA.

**Figure 7:**
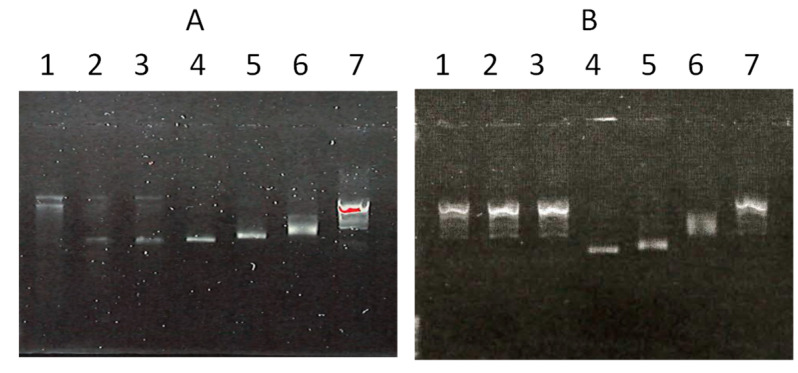
Gel electrophoresis of pIRES2-EGFP plasmid DNA treated with cisplatin, [Cu{Au(Spy)(PTA)}_2_]PF_6_ (**1**) (**A**) and [Au(Spy)(PTA)] (**B**) at metal–DNA base pair ratios of 0.5, 1, and 2. Line 7, untreated plasmid pIRES2-EGFP 24 h incubated at 37 °C; lines 1–3, complex/DNAbp ratios of 2, 1, and 0.5, respectively; lines 4–7 cisplatin/DNAbp ratios of 2, 1, and 0.5, respectively.

In addition, the interaction of both complexes with DNA was also studied by their ability to modify the electrophoretic mobility of the pIRES2-EGFP (5308 pb) plasmid DNA and compared to cisplatin (Figure 7). Treatment with increasing amounts of cisplatin greatly alters the electrophoretic mobility of pIRES2-EGFP. Complex [Au(Spy)(PTA)] does not affect the mobility of the plasmid; however, the heteronuclear derivative [Cu{Au(Spy)(PTA)}_2_]PF_6_ (**1**) produces a significant effect on the mobility of the plasmid, similarly to cisplatin. The lack of reactivity with the DNA of [Au(Spy)(PTA)] is comparable to that observed for auranofin and in previously reported gold(I) derivatives [63,87]. The different behaviour observed in the trinuclear complex is consistent with the very high value of the calculated interaction constant with DNA (9.63 × 10^6^ M^−1^), and the presence of copper in the structure of the complex suggests that this metal is responsible for the interaction [88].

## 4. Conclusions

The synthesis and characterisation of the trinuclear complexes [Cu{Au(Spy)(PTA)}_2_]PF_6_ (**1**), [Zn{Au(Spy)(PTA)}_2_](NO_3_)_2_ (**2**), and [Zn{Au(Spy)(PTA)}_2_]Cl_2_ (**3**) contribute noticeably to the existing literature on gold(I) metalloligands. These heteronuclear complexes show different NMR and IR spectra compared to their mononuclear precursor, indicating the successful coordination of copper(I) and zinc(II) ions. The ability of these complexes to remain stable under physiological conditions likely points to their potential use in biological systems, especially complex **2**. Both complexes **1** and **2** notably show substantial binding affinity to bovine serum albumin, which is essential for their pharmacokinetic properties.

The biological studies reveal that these complexes exhibit highly cytotoxic effects on Caco-2 cancer cell lines, and their activity is enhanced in complex **1** compared to the mononuclear precursor, with significantly lowered IC_50_ values. This enhanced cytotoxicity is attributed to the improved cellular uptake and increased lipophilicity of complex **1**. Furthermore, complex **1** potently induces apoptosis and disrupts the cell cycle in Caco-2 cells, arresting cell proliferation and increasing the proportion of cells undergoing apoptosis.

They further confirm the findings on the increase in intracellular redox states, noticing that both complexes, especially complex **1**, increase ROS levels, which could be the consequence of the inhibition of TrxR. This disruption to redox homeostasis contributes to the cytotoxic effects observed. Additionally, the binding study with ct-DNA and the mobility study with pIRES2-EGFP plasmid DNA revealed a strong affinity of complex **1** for DNA, suggesting potential DNA damage.

In conclusion, this study supports the potential of these heteronuclear gold(I) complexes as effective anticancer agents, with complex **1** showing promising results in inducing apoptosis, cell cycle disruption, DNA interactions, and ROS production in cancer cells. Further studies should focus on elucidating the mechanisms of action, optimising the complexes for better efficacy and selectivity, and evaluating their in vivo therapeutic potential.

## Figures and Tables

**Table 1:** Shift *δ* (ppm) of the ^1^H NMR spectra (DMSO-d_6_) of mono- and heteronuclear complexes.

Complex	H^6^	H^5^	H^4^, H^3^	NCH_2_N (AB System)	NCH_2_P
[Au(Spy)(PTA)]	8.14 (dt)	6.86 (ddd)	7.35 (m)	4.52 and 4.34	4.35 (s)
[Cu{Au(Spy)(PTA)}_2_]PF_6_ (**1**)	8.42 (br s)	7.09 (brs)	7.54 (br s)	4.43 and 4.34	4.25 (s)
[Zn{Au(PTA)(Spy)}_2_](NO_3_)_2_ (**2**)	8.13 (d)	6.87 (ddd)	7.36 (td), 7.29 (d)	4.53 and 4.35	4.35 (s)
[Zn{Au(PTA)(Spy)}_2_]Cl_2_ (**3**)	8.13 (d)	6.87 (ddd)	7.36 (td), 7.30 (d)	4.53 and 4.35	4.35 (s)

**Table 2:** Most significant IR bands (cm^−1^) of homo- and heteronuclear complexes.

Complex	Spy				
	υ(C=C), υ(C=N)	Ring Deformation	PTA	Au-S	Au-P
[Au(Spy)(PTA)]	1648, 1570, 1548	1121, 783, 625	1440, 1409	289	271
**1**	1635, 1570(sh), 1550 1583	1127, 756, 649	1452, 1412	283	270
**2**	1640, 1570(sh), 1548 1590	1137, 758, 648	1451, 1410	288	271
**3**	1640, 1571(sh), 1548 1589	1138, 759, 647	1449, 1410	292	276

**Table 3:** Values of Stern−Volmer quenching constant (K_SV_), the number of binding sites (n) and the apparent binding constant (K_b_) for the interaction of complexes **2** and **3** with BSA.

Complex	K_SV_ (M^−1^)	K_b_ (M^−1^)	n
[Cu{Au(Spy)(PTA)}_2_]PF_6_ (**1**)	5.73 × 10^3^	1.37 × 10^4^	1.11
[Zn{Au(PTA)(Spy)}_2_](NO_3_)_2_ (**2**)	1.76 × 10^3^	1.71 × 10^3^	0.95

**Table 4:** Distribution coefficients and IC_50_ (μM) values of the complexes on Caco-2/PD7 and Caco-2/TC7cells.

Complex	IC_50_ (μM) [a]		
	Caco-2/PD7	Caco-2/TC7	logP
[Au(Spy)(PTA)]	2.64 ± 0.25	2.03 ± 0.02	
[Cu{Au(Spy)(PTA)}_2_]PF_6_ (**1**)	0.16 ± 0.02	0.13 ± 0.07	0.84
[Zn{Au(PTA)(Spy)}_2_](NO_3_)_2_ (**2**)	2.18 ± 0.24	1.94 ± 0.81	0.47
Auranofin	1.8 ± 0.1	2.1 ± 0.4	−2.53

[a] Mean ± SE of at least three determinations.

## Data Availability

Data are contained within the article and Supplementary Materials.

## Supplementary Materials

The following are available online at https://www.mdpi.com/article/10.3390/biomedicines12081763/s1, Figures S1–S12: ^1^H, ^31^P{^1^H} and HSQC NMR spectra of heteronuclear complexes, Figure S13: 1H NMR spectra of complex 3 at different temperatures, Figures S14–S16: Cell viability, Figure S17. Density plots from the analysis of Caco-2/TC7 and Caco-2/PD7 cells, Figures S18 and S20. Electronic absorption spectra of complex **1**,**2** in the absence and the presence of ct-DNA, Figures S19 and S21. Plot of [DNA]/[εa − εf] vs. [DNA]. Figure S22. Stern−Volmer plots. Figure S23. Representation of the modified Stern−Volmer plot.

## References

[1] Sung H., Ferlay J., Siegel R.L., Laversanne M., Soerjomataram I., Jemal A., Bray F. (2021). Global Cancer Statistics 2020: GLOBOCAN Estimates of Incidence and Mortality Worldwide for 36 Cancers in 185 Countries. CA Cancer J. Clin..

[2] Ahmed S., Johnson K., Ahmed O., Iqbal N. (2014). Advances in the management of colorectal cancer: From biology to treatment. Int. J. Color. Dis..

[3] Adebayo A.S., Agbaje K., Adesina S.K., Olajubutu O. (2023). Colorectal Cancer: Disease Process, Current Treatment Options, and Future Perspectives. Pharmaceutics.

[4] Casini A., Pöthig A. (2024). Metals in Cancer Research: Beyond Platinum Metallodrugs. ACS Cent. Sci..

[5] Abdolmaleki S., Aliabadi A., Khaksar S. (2024). Riding the metal wave: A review of the latest developments in metal-based anticancer agents. Coord. Chem. Rev..

[6] Yeo C.I., Goh C.H.P., Tiekink E.R.T., Chew J. (2024). Antibiotics: A “GOLDen” promise?. Coord. Chem. Rev..

[7] Stratton M., Ramachandran A., Camacho E.J.M., Patil S., Waris G., Grice K.A. (2020). Anti-fibrotic activity of gold and platinum complexes—Au(I) compounds as a new class of anti-fibrotic agents. J. Inorg. Biochem..

[8] Mertens R.T., Gukathasan S., Arojojoye A.S., Olelewe C., Awuah S.G. (2023). Next Generation Gold Drugs and Probes: Chemistry and Biomedical Applications. Chem. Rev..

[9] Essa R.Z., Brianna, Yeo C.I., Teow S.-Y. (2024). Gold complexes and their molecular targets in colorectal cancer. J. Organomet. Chem..

[10] Pete S., Roy N., Kar B., Paira P. (2022). Construction of homo and heteronuclear Ru(II), Ir(III) and Re(I) complexes for target specific cancer therapy. Coord. Chem. Rev..

[11] Ma L., Li L., Zhu G. (2022). Platinum-containing heterometallic complexes in cancer therapy: Advances and perspectives. Inorg. Chem. Front..

[12] Giorgi E., Binacchi F., Marotta C., Cirri D., Gabbiani C., Pratesi A. (2023). Highlights of New Strategies to Increase the Efficacy of Transition Metal Complexes for Cancer Treatments. Molecules.

[13] Redrado M., Fernández-Moreira V., Gimeno M.C. (2021). Theranostics Through the Synergistic Cooperation of Heterometallic Complexes. ChemMedChem.

[14] López-Hernández J.E., Nayeem N., Cerón-Carrasco J.P., Ahad A., Hafeez A., León I.E., Contel M. (2023). Platinum(IV)–Gold(I) Agents with Promising Anticancer Activity: Selected Studies in 2D and 3D Triple-Negative Breast Cancer Models. Chem. Eur. J..

[15] Wenzel M., Bigaeva E., Richard P., Le Gendre P., Picquet M., Casini A., Bodio E. (2014). New heteronuclear gold(I)-platinum(II) complexes with cytotoxic properties: Are two metals better than one?. J. Inorg. Biochem..

[16] Serratrice M., Maiore L., Zucca A., Stoccoro S., Landini I., Mini E., Massai L., Ferraro G., Merlino A., Messori L. (2016). Cytotoxic properties of a new organometallic platinum(ii) complex and its gold(i) heterobimetallic derivatives. Dalton Trans..

[17] Bellam R., Jaganyi D., Robinson R.S. (2022). Heterodinuclear Ru–Pt Complexes Bridged with 2,3-Bis(pyridyl)pyrazinyl Ligands: Studies on Kinetics, Deoxyribonucleic Acid/Bovine Serum Albumin Binding and Cleavage, In Vitro Cytotoxicity, and In Vivo Toxicity on Zebrafish Embryo Activities. ACS Omega.

[18] Bellam R., Jaganyi D., Mambanda A., Robinson R. (2018). Role of a 2,3-bis(pyridyl)pyrazinyl chelate bridging ligand in the reactivity of Ru(ii)–Pt(ii) dinuclear complexes on the substitution of chlorides by thiourea nucleophiles—A kinetic study. New J. Chem..

[19] Ma L., Ma R., Wang Z., Yiu S.-M., Zhu G. (2016). Heterodinuclear Pt(iv)–Ru(ii) anticancer prodrugs to combat both drug resistance and tumor metastasis. Chem. Commun..

[20] Ma L., Lin X., Li C., Xu Z., Chan C.Y., Tse M.K., Shi P., Zhu G. (2018). A Cancer Cell-Selective and Low-Toxic Bifunctional Heterodinuclear Pt(IV)-Ru(II) Anticancer Prodrug. Inorg. Chem..

[21] Gonzalez-Pantoja J.F., Stern M., Jarzecki A.A., Royo E., Robles-Escajeda E., Varela-Ramirez A., Aguilera R.J., Contel M. (2011). Titanocene-Phosphine Derivatives as Precursors to Cytotoxic Heterometallic TiAu_2_ and TiM (M = Pd, Pt) Compounds. Studies of Their Interactions with DNA. Inorg. Chem..

[22] Mitra K., Shettar A., Kondaiah P., Chakravarty A.R. (2016). Biotinylated Platinum(II) Ferrocenylterpyridine Complexes for Targeted Photoinduced Cytotoxicity. Inorg. Chem..

[23] Kowalski K. (2018). Recent developments in the chemistry of ferrocenyl secondary natural product conjugates. Coord. Chem. Rev..

[24] Askari B., Rudbari H.A., Micale N., Schirmeister T., Maugeri A., Navarra M. (2019). Anticancer study of heterobimetallic platinum(II)-ruthenium(II) and platinum(II)-rhodium(III) complexes with bridging dithiooxamide ligand. J. Organomet. Chem..

[25] Aranda E.E., da Luz J.S., Oliveira C.C., Divina Petersen P.A., Petrilli H.M., da Costa Ferreira A.M. (2020). Heterobinuclear copper(II)-platinum(II) complexes with oxindolimine ligands: Interactions with DNA, and inhibition of kinase and alkaline phosphatase proteins. J. Inorg. Biochem..

[26] Ćoćić D., Jovanović-Stević S., Jelić R., Matić S., Popović S., Djurdjević P., Baskić D., Petrović B. (2020). Homo- and hetero-dinuclear Pt(ii)/Pd(ii) complexes: Studies of hydrolysis, nucleophilic substitution reactions, DNA/BSA interactions, DFT calculations, molecular docking and cytotoxic activity. Dalton Trans..

[27] Zhang C., Guan R., Liao X., Ouyang C., Liu J., Ji L., Chao H. (2020). Mitochondrial DNA targeting and impairment by a dinuclear Ir–Pt complex that overcomes cisplatin resistance. Inorg. Chem. Front..

[28] Vučelj S., Hasić R., Ašanin D., Šmit B., Caković A., Bogojeski J., Serafinović M.Ć., Marković B.S., Stojanović B., Pavlović S. (2024). Modes of Interactions with DNA/HSA Biomolecules and Comparative Cytotoxic Studies of Newly Synthesized Mononuclear Zinc(II) and Heteronuclear Platinum(II)/Zinc(II) Complexes toward Colorectal Cancer Cells. Int. J. Mol. Sci..

[29] Yousuf I., Bashir M., Arjmand F., Tabassum S. (2021). Advancement of metal compounds as therapeutic and diagnostic metallodrugs: Current frontiers and future perspectives. Coord. Chem. Rev..

[30] Chandra A., Singh K., Singh S., Sivakumar S., Patra A.K. (2016). A luminescent europium(iii)–platinum(ii) heterometallic complex as a theranostic agent: A proof-of-concept study. Dalton Trans..

[31] Elie B.T., Fernández-Gallardo J., Curado N., Cornejo M.A., Ramos J.W., Contel M. (2019). Bimetallic titanocene-gold phosphane complexes inhibit invasion, metastasis, and angiogenesis-associated signaling molecules in renal cancer. Eur. J. Med. Chem..

[32] Elie B.T., Pechenyy Y., Uddin F., Contel M. (2018). A heterometallic ruthenium-gold complex displays antiproliferative, antimigratory, and antiangiogenic properties and inhibits metastasis and angiogenesis-associated proteases in renal cancer. J. Biol. Inorg. Chem..

[33] Massai L., Fernandez-Gallardo J., Guerri A., Arcangeli A., Pillozzi S., Contel M., Messori L. (2015). Design, synthesis and characterisation of new chimeric ruthenium(II)-gold(I) complexes as improved cytotoxic agents. Dalton Trans..

[34] Lease N., Vasilevski V., Carreira M., de Almeida A., Sanau M., Hirva P., Casini A., Contel M. (2013). Potential Anticancer Heterometallic Fe-Au and Fe-Pd Agents: Initial Mechanistic Insights. J. Med. Chem..

[35] Garcia-Moreno E., Gascon S., Rodriguez-Yoldi M.J., Cerrada E., Laguna M. (2013). S-Propargylthiopyridine Phosphane Derivatives As Anticancer Agents: Characterization and Antitumor Activity. Organometallics.

[36] Johnson A., Marzo I., Gimeno M.C. (2020). Heterobimetallic propargyl gold complexes with pi-bound copper or silver with enhanced anticancer activity. Dalton Trans..

[37] Kaim W., Schwederski B., Klein A. (2013). Bioinorganic Chemistry--Inorganic Elements in the Chemistry of Life: An Introduction and Guide.

[38] Tisato F., Marzano C., Porchia M., Pellei M., Santini C. (2010). Copper in diseases and treatments, and copper-based anticancer strategies. Med. Res. Rev..

[39] Lelièvre P., Sancey L., Coll J.-L., Deniaud A., Busser B. (2020). The Multifaceted Roles of Copper in Cancer: A Trace Metal Element with Dysregulated Metabolism, but Also a Target or a Bullet for Therapy. Cancers.

[40] Wang Y., Tang T., Yuan Y., Li N., Wang X., Guan J. (2024). Copper and Copper Complexes in Tumor Therapy. ChemMedChem.

[41] Pellei M., Del Bello F., Porchia M., Santini C. (2021). Zinc coordination complexes as anticancer agents. Coord. Chem. Rev..

[42] Vergara E., Casini A., Sorrentino F., Zava O., Cerrada E., Rigobello M.P., Bindoli A., Laguna M., Dyson P.J. (2010). Anticancer Therapeutics That Target Selenoenzymes: Synthesis, Characterization, in vitro Cytotoxicity, and Thioredoxin Reductase Inhibition of a Series of Gold(I) Complexes Containing Hydrophilic Phosphine Ligands. ChemMedChem.

[43] Miranda S., Vergara E., Mohr F., de Vos D., Cerrada E., Mendia A., Laguna M. (2008). Synthesis, characterization, and in vitro cytotoxicity of some gold(I) and trans platinum(II) thionate complexes containing water-soluble PTA and DAPTA ligands. X-ray crystal structures of Au(SC_4_H3N_2_)(PTA), trans-Pt(SC_4_H_3_N_2_)_2_(PTA)_2_, trans-Pt(SC5H_4_N)_2_(PTA)_2_, and trans-Pt(SC5H_4_N)_2_(DAPTA)_2_. Inorg. Chem..

[44] Daigle D.J., Pepperman A.B., Vail S.L. (1974). Synthesis of a monophosphorus analog of hexamethylenetetramine. J. Hetercycl. Chem..

[45] Atrián-Blasco E., Gascón S., Rodríguez-Yoldi M.J., Laguna M., Cerrada E. (2016). Synthesis of Gold(I) Derivatives Bearing Alkylated 1,3,5-Triaza-7-phosphaadamantane as Selective Anticancer Metallodrugs. Eur. J. Inorg. Chem..

[46] Pinto M., Robineleon S., Appay M.D., Kedinger M., Triadou N., Dussaulx E., Lacroix B., Simonassmann P., Haffen K., Fogh J. (1983). Enterocyte-like differentiation and polarization of the human-colon carcinoma cell-line caco-2 in culture. Biol. Cell.

[47] Chantret I., Rodolosse A., Barbat A., Dussaulx E., Brotlaroche E., Zweibaum A., Rousset M. (1994). Differential Expression Of Sucrase-Isomaltase In Clones Isolated From Early And Late Passages Of The Cell-Line Caco-2—Evidence For Glucose-Dependent Negative Regulation. J. Cell Sci..

[48] Marmol I., Virumbrales-Munoz M., Quero J., Sanchez-De-Diego C., Fernandez L., Ochoa I., Cerrada E., Yoldi M.J.R. (2017). Alkynyl gold(I) complex triggers necroptosis via ROS generation in colorectal carcinoma cells. J. Inorg. Biochem..

[49] Vergara E., Miranda S., Mohr F., Cerrada E., Tiekink E.R.T., Romero P., Mendia A., Laguna M. (2007). Gold(I) and Palladium(II) thiolato complexes containing water-soluble phosphane ligands. Eur. J. Inorg. Chem..

[50] Hashimoto Y., Yoshinari N., Naruse D., Nozaki K., Konno T. (2013). Synthesis, Structures, and Luminescence Properties of Interconvertible AuI_2_ZnII and AuI_3_ZnII Complexes with Mixed Bis(diphenylphosphino)methane and d-Penicillaminate. Inorg. Chem..

[51] Smoleński P., Benisvy L., Guedes da Silva M.F.C., Pombeiro A.J.L. (2009). Syntheses and Crystal Structures of the First Zinc Complex with 1,3,5-Triaza-7-phosphaadamantane (PTA), [ZnCl_2_(PTA)_2_], and of the Hybrid Organic–Inorganic Salts of N-Methyl-1,3,5-triaza-7-phosphaadamantane with Tetrahalozinc [PTA–Me]_2_ [ZnI_2_X_2_] (X = I, Cl). Eur. J. Inorg. Chem..

[52] Spinner E. (1960). 250. The infrared spectra of some N-heteroaromatic mercaptocompounds and of their N-methyl and S-methyl derivatives. J. Chem. Soc..

[53] Sousa-Pedrares A., Romero J., Arturo García-Vázquez J., Luz Durán M., Casanova I., Sousa A. (2003). Electrochemical synthesis and structural characterisation of zinc, cadmium and mercury complexes of heterocyclic bidentate ligands (N, S). Dalton Trans..

[54] Rodríguez A., Sousa-Pedrares A., García-Vázquez J.A., Romero J., Sousa A. (2011). Synthesis and Structural Characterization of Copper(I), Silver(I) and Gold(I) Complexes with Pyrimidine-2-thionato Ligands and their Adducts with Phosphanes. Eur. J. Inorg. Chem..

[55] Radisavljević S., Petrović B. (2020). Gold(III) Complexes: An Overview on Their Kinetics, Interactions With DNA/BSA, Cytotoxic Activity, and Computational Calculations. Front. Chem..

[56] Eftink M. (2006). Fluorescence Techniques for Studying Protein Structure. Methods of Biochemical Analysis: Protein Structure Determination.

[57] Lakowicz J.R. (2006). Principles of Fluorescence Spectroscopy.

[58] Galassi R., Luciani L., Gambini V., Vincenzetti S., Lupidi G., Amici A., Marchini C., Wang J.B., Pucciarelli S. (2021). Multi-Targeted Anticancer Activity of Imidazolate Phosphane Gold(I) Compounds by Inhibition of DHFR and TrxR in Breast Cancer Cells. Front. Chem..

[59] Binacchi F., Guarra F., Cirri D., Marzo T., Pratesi A., Messori L., Gabbiani C., Biver T. (2020). On the Different Mode of Action of Au(I)/Ag(I)-NHC Bis-Anthracenyl Complexes Towards Selected Target Biomolecules. Molecules.

[60] Atrian-Blasco E., Gascon S., Rodriguez-Yoldi M.J., Laguna M., Cerrada E. (2017). Novel Gold(I) Thiolate Derivatives Synergistic with 5-Fluorouracil as Potential Selective Anticancer Agents in Colon Cancer. Inorg. Chem..

[61] Kharpan B., Shyam A., Nandi R., Paul S., Paul P.C., Mondal P., Kumar D., Choudhury S., Ray S. (2024). Antiparasitic activity, DNA/BSA binding interaction, molecular docking and DFT studies of mesogenic L-leucine based Schiff base and its derivatize Cu(II) and Zn(II) complexes. J. Mol. Struct..

[62] Ansari M.F., Arjmand F. (2024). Synthesis and characterization of copper(II) norcraugsodine complexes: Their in vitro binding studies with therapeutic targets (ct-DNA/tRNA/BSA), cleavage, and cytotoxicity profile. J. Mol. Struct..

[63] Garcia-Moreno E., Gascon S., Atrian-Blasco E., Rodriguez-Yoldi M.J., Cerrada E., Laguna M. (2014). Gold(I) complexes with alkylated PTA (1,3,5-triaza-7-phosphaadamantane) phosphanes as anticancer metallodrugs. Eur. J. Med. Chem..

[64] Abas E., Pena-Martinez R., Aguirre-Ramírez D., Rodriguez-Dieguez A., Laguna M., Grasa L. (2020). New selective thiolate gold(i) complexes inhibit the proliferation of different human cancer cells and induce apoptosis in primary cultures of mouse colon tumors. Dalton Trans..

[65] Moal V.L.L., Servin A.L. (2013). Pathogenesis of Human Enterovirulent Bacteria: Lessons from Cultured, Fully Differentiated Human Colon Cancer Cell Lines. Microbiol. Mol. Biol. Rev..

[66] Perillo B., Di Donato M., Pezone A., Di Zazzo E., Giovannelli P., Galasso G., Castoria G., Migliaccio A. (2020). ROS in cancer therapy: The bright side of the moon. Exp. Mol. Med..

[67] Trachootham D., Alexandre J., Huang P. (2009). Targeting cancer cells by ROS-mediated mechanisms: A radical therapeutic approach?. Nat. Rev. Drug Discov..

[68] Bhatti J.S., Bhatti G.K., Reddy P.H. (2017). Mitochondrial dysfunction and oxidative stress in metabolic disorders—A step towards mitochondria based therapeutic strategies. Biochim. Biophys. Acta (BBA) Mol. Basis Dis..

[69] Hasan A.A., Kalinina E., Tatarskiy V., Shtil A. (2022). The Thioredoxin System of Mammalian Cells and Its Modulators. Biomedicines.

[70] Arnér E.S.J., Schmidt H.H.H.W., Ghezzi P., Cuadrado A. (2021). Effects of Mammalian Thioredoxin Reductase Inhibitors. Reactive Oxygen Species: Network Pharmacology and Therapeutic Applications.

[71] Lu Y., Zhao X., Li K., Luo G., Nie Y., Shi Y., Zhou Y., Ren G., Feng B., Liu Z. (2013). Thioredoxin-like protein 2 is overexpressed in colon cancer and promotes cancer cell metastasis by interaction with ran. Antioxid. Redox Signal..

[72] Jia J.-J., Geng W.-S., Wang Z.-Q., Chen L., Zeng X.-S. (2019). The role of thioredoxin system in cancer: Strategy for cancer therapy. Cancer Chemother. Pharmacol..

[73] Muangthong T., Chusangnin P., Hassametto A., Tanomrat R., Suwannalert P. (2022). Thioredoxin Reductase-1 as a Potential Biomarker in Fibroblast-Associated HCT116 Cancer Cell Progression and Dissemination in a Zebrafish Model. Cancers.

[74] Shen X., Xia Y., Lu H., Zheng P., Wang J., Chen Y., Xu C., Qiu C., Zhang Y., Xiao Z. (2024). Synergistic targeting of TrxR1 and ATM/AKT pathway in human colon cancer cells. Biomed. Pharmacother..

[75] Zhang J.M., Li X.M., Han X., Liu R.J., Fang J.G. (2017). Targeting the Thioredoxin System for Cancer Therapy. Trends Pharmacol. Sci..

[76] Marmol I., Montanel-Perez S., Royo J.C., Gimeno M.C., Villacampa M.D., Rodriguez-Yoldi M.J., Cerrada E. (2020). Gold(I) and Silver(I) Complexes with 2-Anilinopyridine-Based Heterocycles as Multitarget Drugs against Colon Cancer. Inorg. Chem..

[77] Marmol I., Sanchez-De-Diego C., Dieste A.P., Cerrada E., Yoldi M.J.R. (2017). Colorectal Carcinoma: A General Overview and Future Perspectives in Colorectal Cancer. Int. J. Mol. Sci..

[78] Quero J., Royo J.C., Fodor B., Gimeno M.C., Osada J., Rodríguez-Yoldi M.J., Cerrada E. (2022). Sulfonamide-Derived Dithiocarbamate Gold(I) Complexes Induce the Apoptosis of Colon Cancer Cells by the Activation of Caspase 3 and Redox Imbalance. Biomedicines.

[79] Zhang X.N., Selvaraju K., Saei A.A., D’Arcy P., Zubarev R.A., Arner E.S.J., Linder S. (2019). Repurposing of auranofin: Thioredoxin reductase remains a primary target of the drug. Biochimie.

[80] Zeglis B.M., Pierre V.C., Barton J.K. (2007). Metallo-intercalators and metallo-insertors. Chem. Commun..

[81] Pages B.J., Ang D.L., Wright E.P., Aldrich-Wright J.R. (2015). Metal complex interactions with DNA. Dalton Trans..

[82] Ali I., Wani W.A., Saleem K., Hsieh M.-F. (2014). Anticancer metallodrugs of glutamic acid sulphonamides: In silico, DNA binding, hemolysis and anticancer studies. RSC Adv..

[83] Sirajuddin M., Ali S., Badshah A. (2013). Drug-DNA interactions and their study by UV-Visible, fluorescence spectroscopies and cyclic voltametry. J. Photochem. Photobiol. B.

[84] Radisavljevic S., Cocic D., Jovanovic S., Smit B., Petkovic M., Milivojevic N., Planojevic N., Markovic S., Petrovic B. (2019). Synthesis, characterization, DFT study, DNA/BSA-binding affinity, and cytotoxicity of some dinuclear and trinuclear gold(III) complexes. J. Biol. Inorg. Chem..

[85] Alsaeedi M.S., Babgi B.A., Hussien M.A., Abdellattif M.H., Humphrey M.G. (2020). DNA-Binding and Anticancer Activity of Binuclear Gold(I) Alkynyl Complexes with a Phenanthrenyl Bridging Ligand. Molecules.

[86] Dimiza F., Perdih F., Tangoulis V., Turel I., Kessissoglou D.P., Psomas G. (2011). Interaction of copper(II) with the non-steroidal anti-inflammatory drugs naproxen and diclofenac: Synthesis, structure, DNA- and albumin-binding. J. Inorg. Biochem..

[87] Mirabelli C.K., Sung C.M., Zimmerman J.P., Hill D.T., Mong S., Crooke S.T. (1986). Interactions of gold coordination-complexes with DNA. Biochem. Pharmacol..

[88] Pivetta T., Valletta E., Ferino G., Isaia F., Pani A., Vascellari S., Castellano C., Demartin F., Cabiddu M.G., Cadoni E. (2017). Novel coumarins and related copper complexes with biological activity: DNA binding, molecular docking and in vitro antiproliferative activity. J. Inorg. Biochem..

