# Supplementary material for: Heteronuclear Complexes with Promising Anticancer Activity against Colon Cancer
Source: Biomedicines. 2024 Aug 5;12(8):1763. doi: 10.3390/biomedicines12081763 (PMC11351612; doi:10.3390/biomedicines12081763)
Supplement: Supplementary file 1 [file biomedicines-12-01763-s001.zip › biomedicines-3126041-supplementary.pdf]

Heteronuclear complexes with promising anticancer activity against colon cancer

Elena Atrián-Blasco,[1†] Javier Saez,[1] María Jesús Rodriguez-Yoldi[2] and Elena Cerrada,[1*]

[1] Departamento de Química Inorgánica, Instituto de Síntesis Química y Catálisis Homogénea-ISQCH, Universidad de Zaragoza-C.S.I.C., 50009 Zaragoza, Spain; ecerrada@unizar.es, j.saez@unizar.es
[2] Departamento de Farmacología y Fisiología, Medicina Legal y Forense. Unidad de Fisiología, Facultad de Veterinaria, Ciber de Fisiopatología de la Obesidad y Nutrición (CIBERobn), Instituto Agroalimentario de Aragón (IA2), 50013, Zaragoza, Spain and Instituto de Investigación Sanitaria de Aragón (IIS Aragón), 50009, Zaragoza, Spain; mjrodyol@unizar.es
* Correspondence: ecerrada@unizar.es and mjrodyol@unizar.es
† Current affiliation: Instituto de Nanociencia y Materiales de Aragón (INMA), CSIC-Universidad de Zaragoza, 50009 Zaragoza, Spain

Table of Contents

Figures S1-S13. NMR spectra of the compounds.

Figure S14. UV-Vis spectra of the complexes

Figure S15. % Cell viability-concentration plot for the treatment of Caco-2 cells with complexes

Figure S16. Cell viability of differentiated Caco-2 cells.

Figure S17. Density plots.

Figures S18 and S20. Electronic absorption spectra of complexes in the absence and the presence of increasing amounts of ct-DNA.

Figures S19 and S21. Plots of [DNA]/[εa − εf] vs. [DNA]

Figure S22. Stern−Volmer plots

Figure 23. Representation of the modified Stern−Volmer plot.

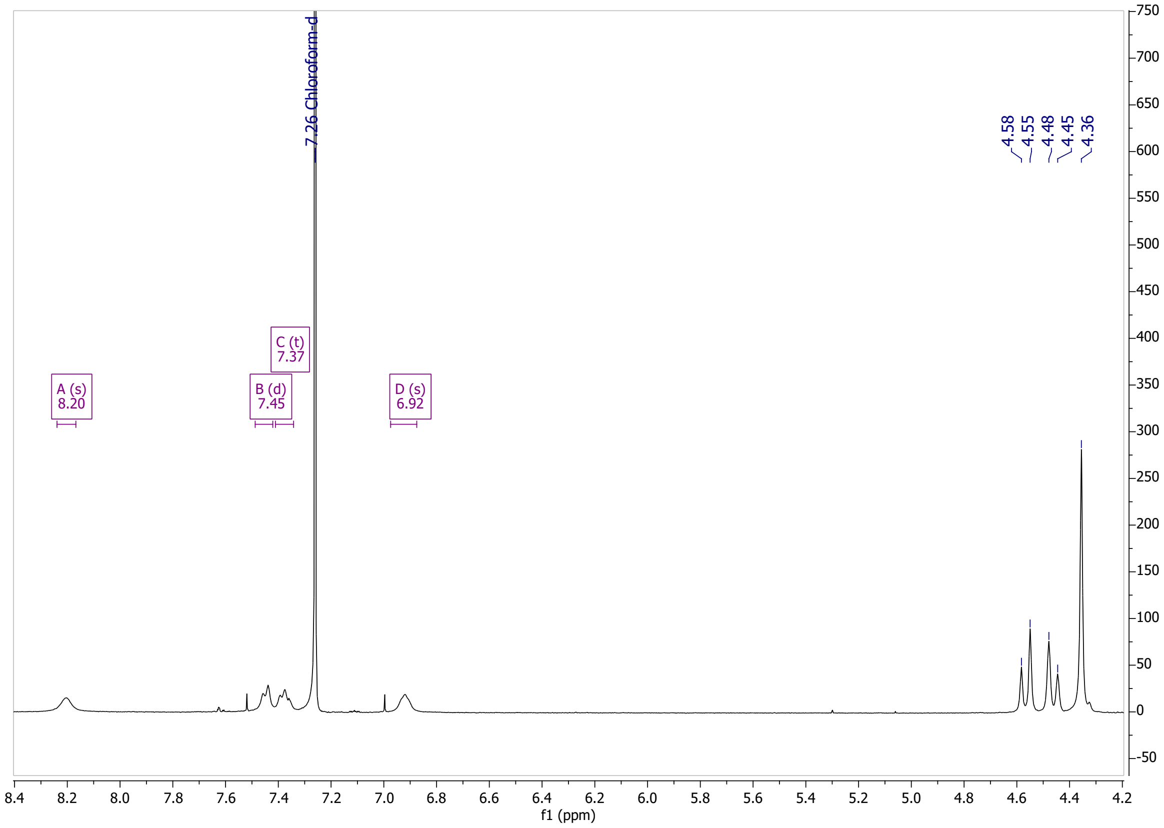

Figure S1. ^{1}H NMR spectrum of complex **2** in $CDCl_3$.

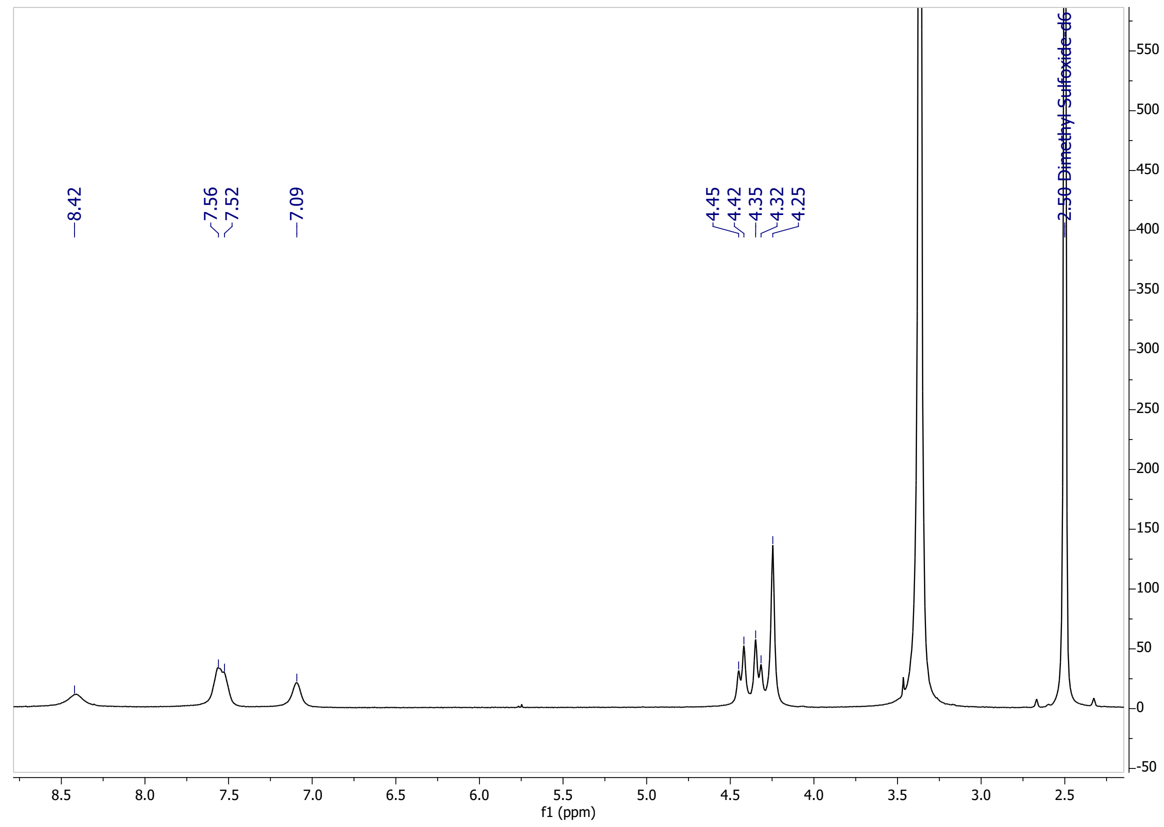

Figure S2. ^{1}H NMR spectrum of complex **2** in DMSO-d_6.

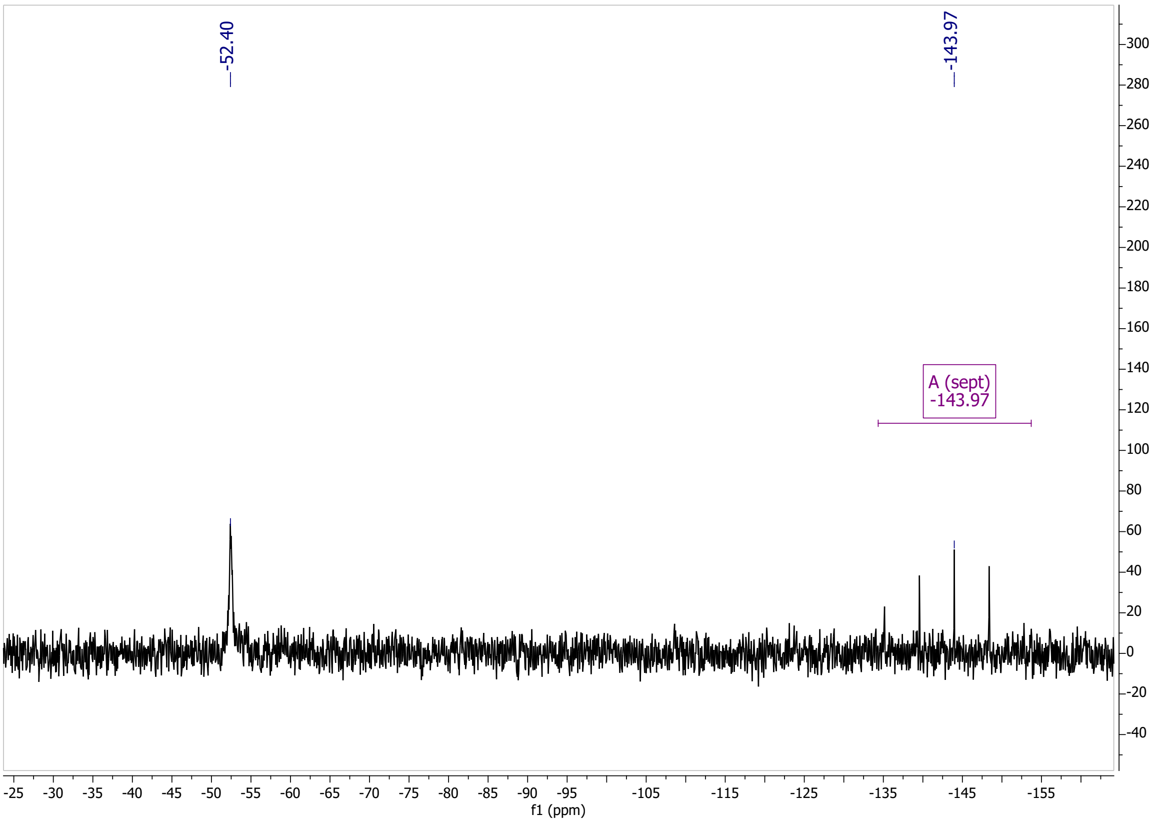

Figure S3. ^{31}P NMR spectrum of complex **2**.

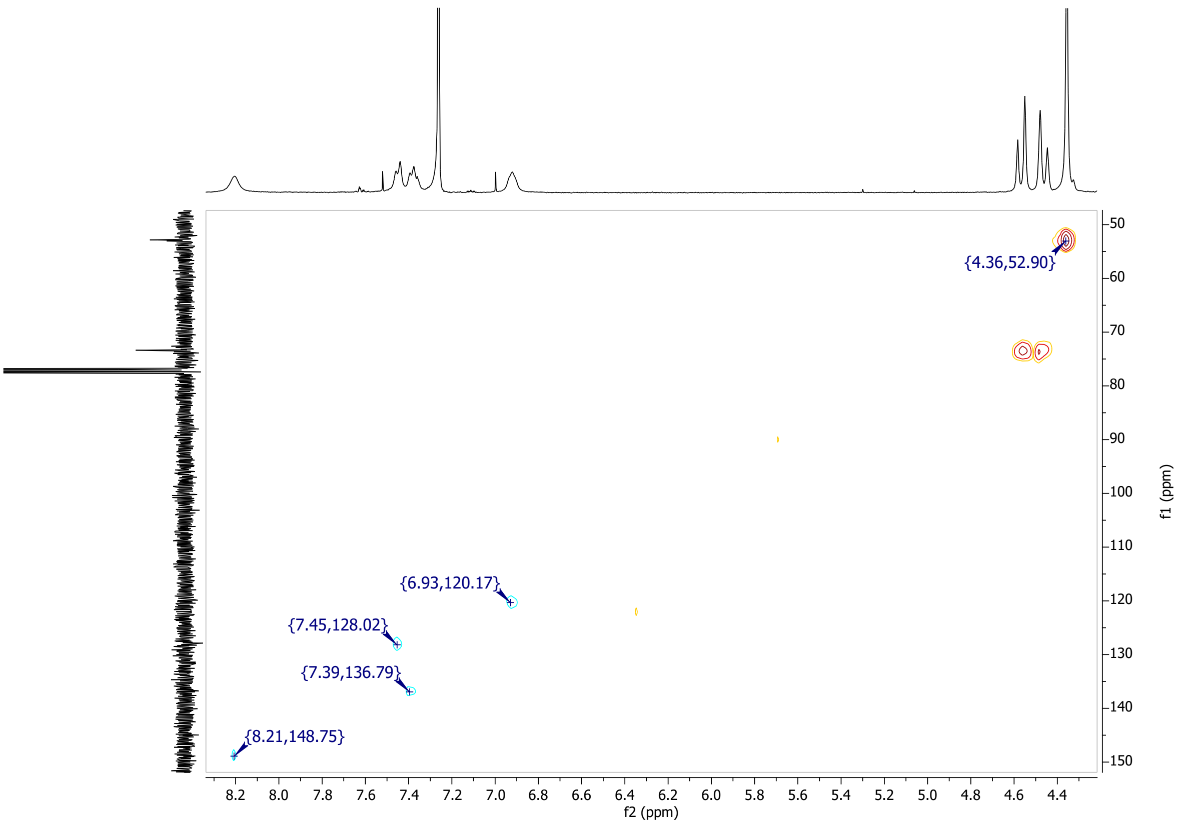

Figure S4. HSQC NMR spectrum of complex **2**.

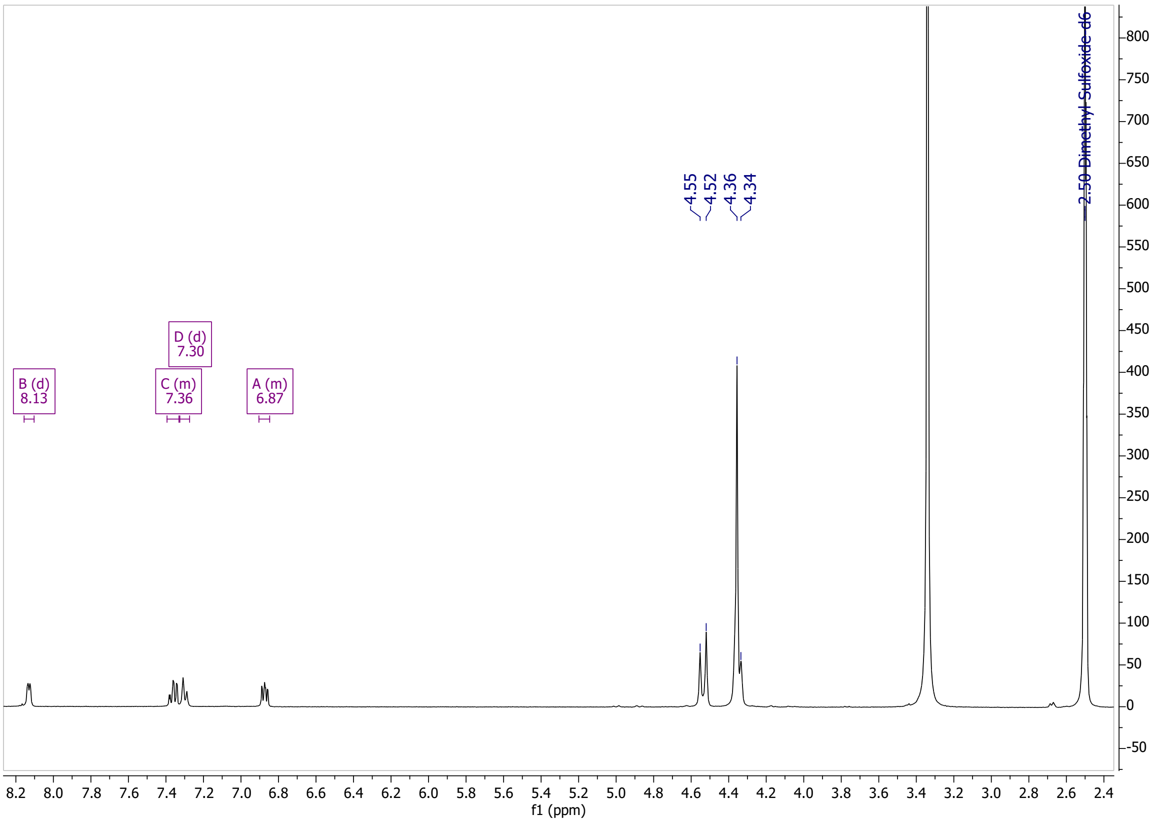

Figure S5. ^{1}H NMR spectrum of complex **3** in DMSO-d_6.

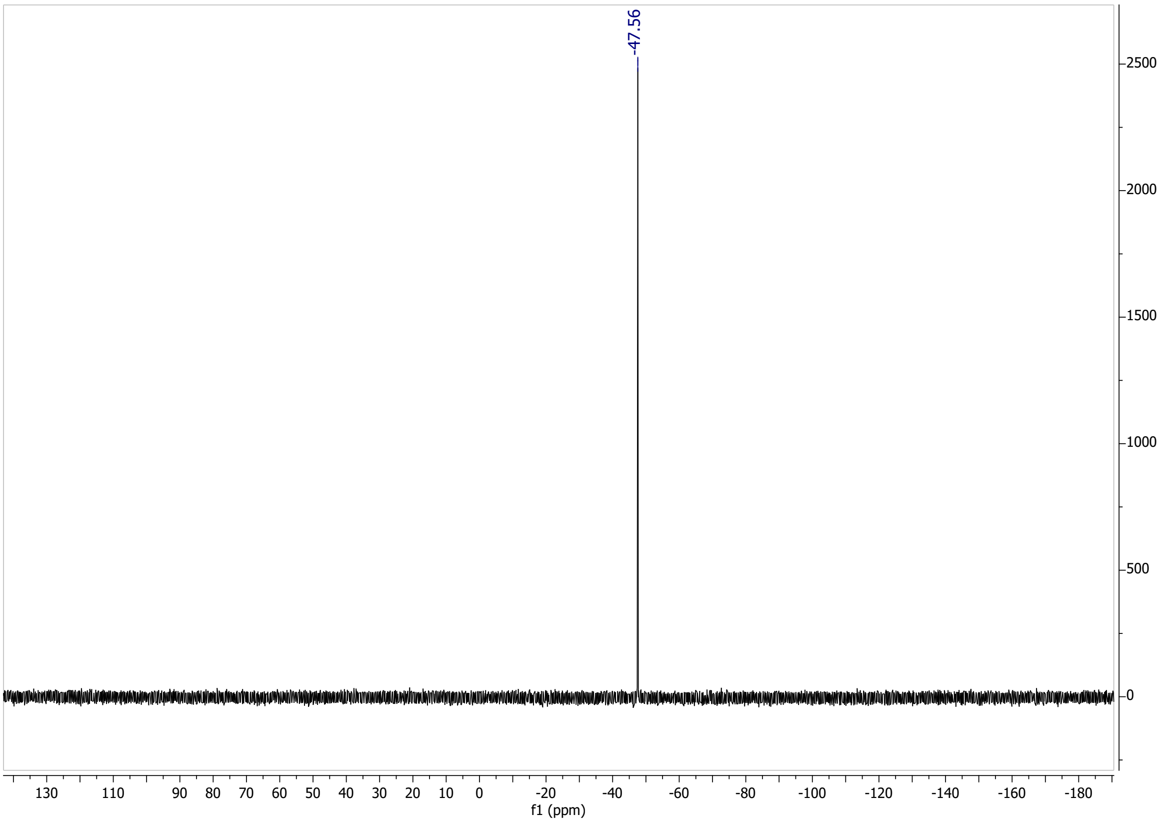

Figure S6. ^{31}P NMR spectrum of complex **3**.

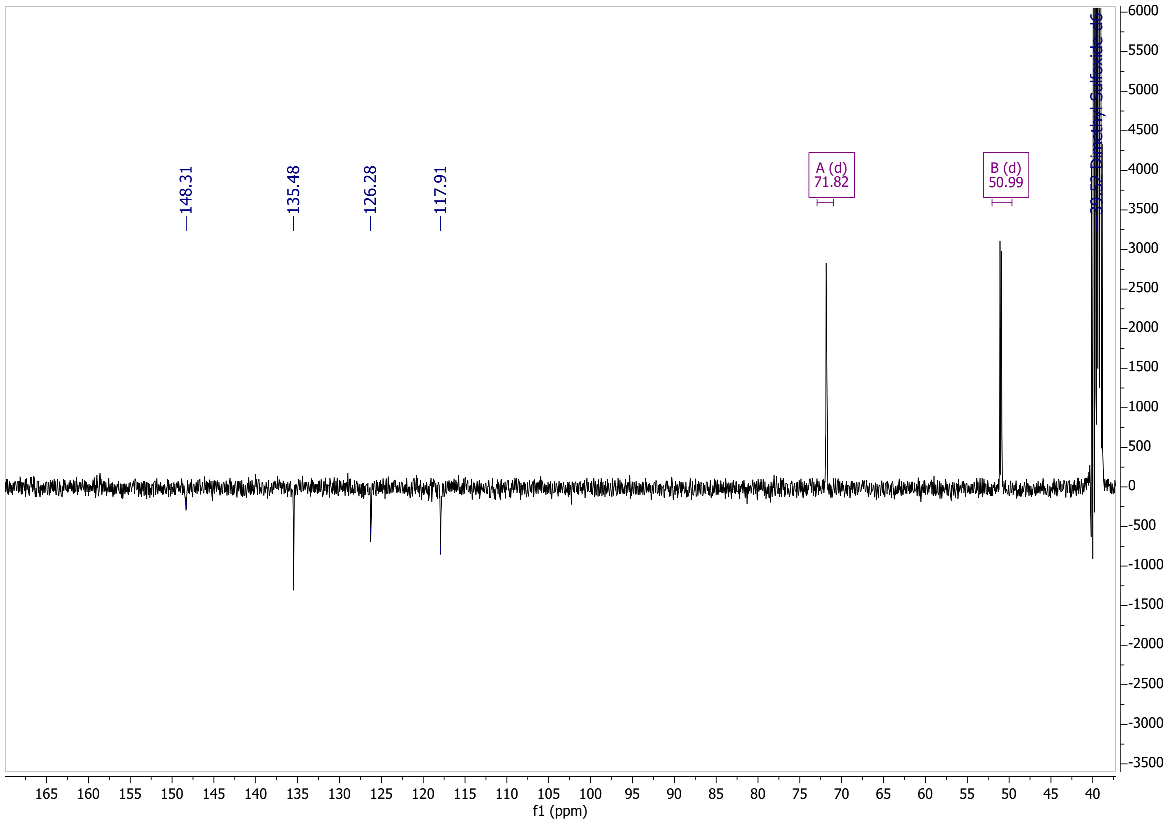

Figure S7. ^{13}C NMR spectrum of complex **3**.

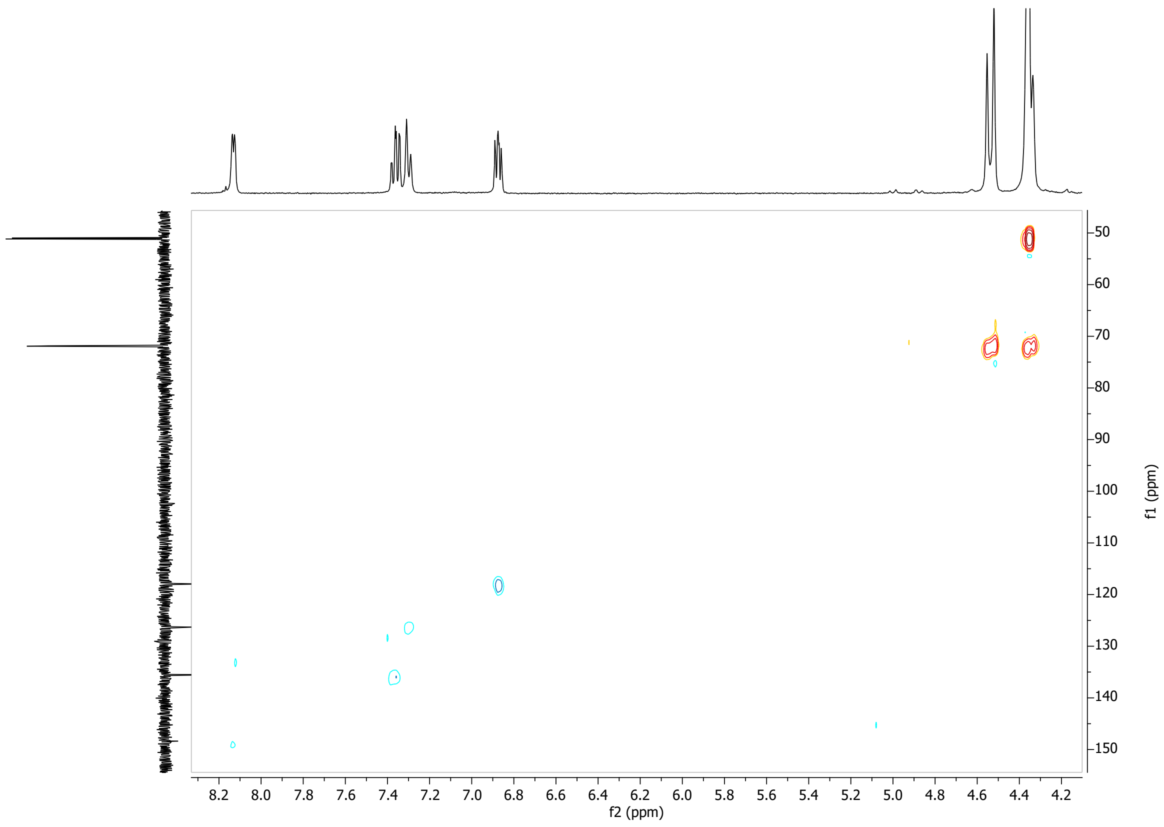

Figure S8. HSQC NMR spectrum of complex **3**.

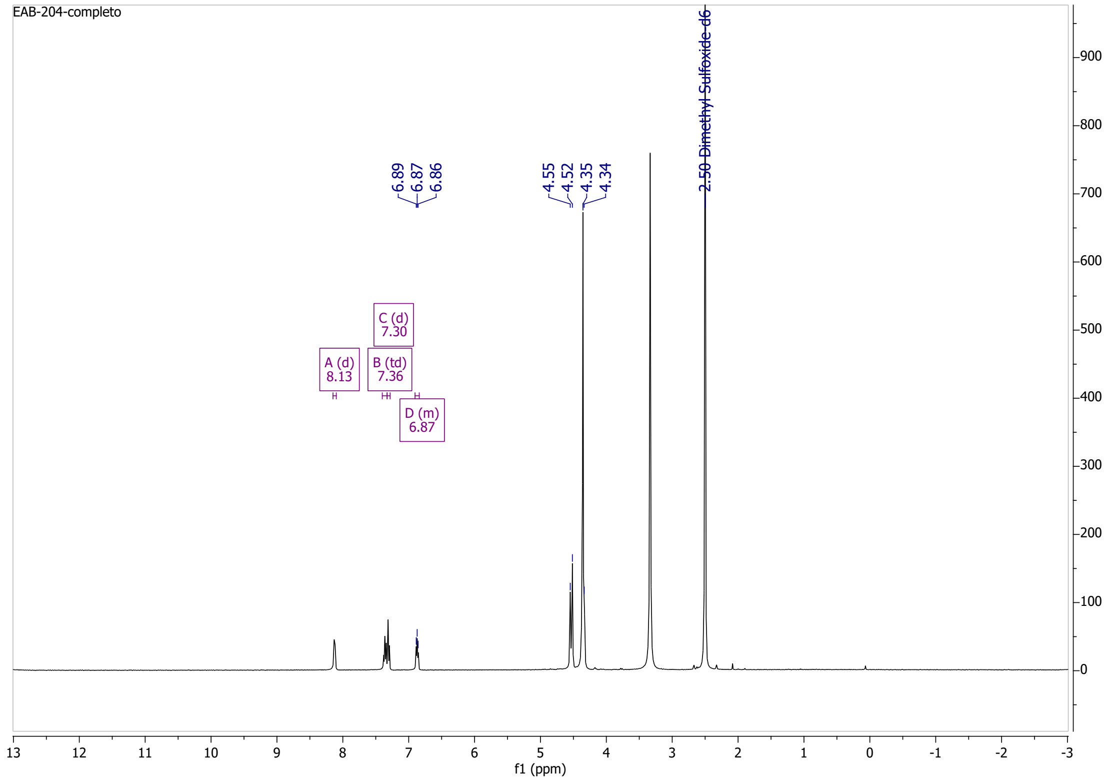

Figure S9. ^{1}H NMR spectrum of complex **4** in DMSO-d_6.

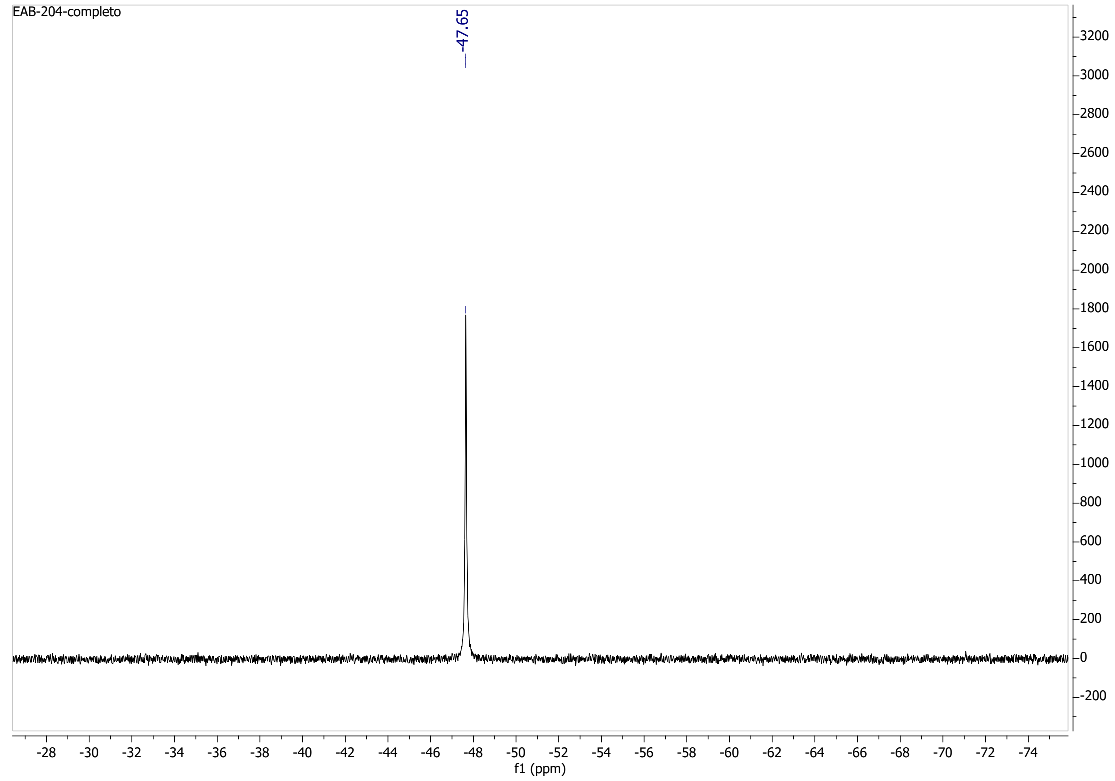

Figure S10. ^{31}P NMR spectrum of complex **4**.

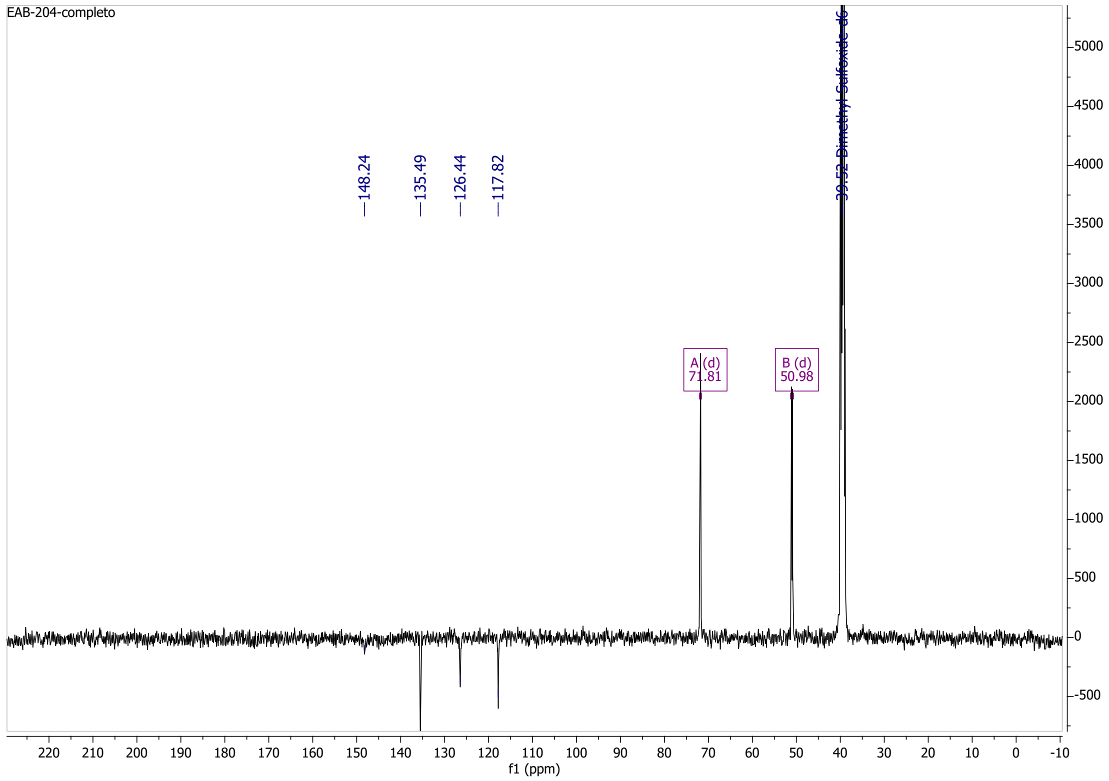

Figure S11. ^{13}C NMR spectrum of complex **4**.

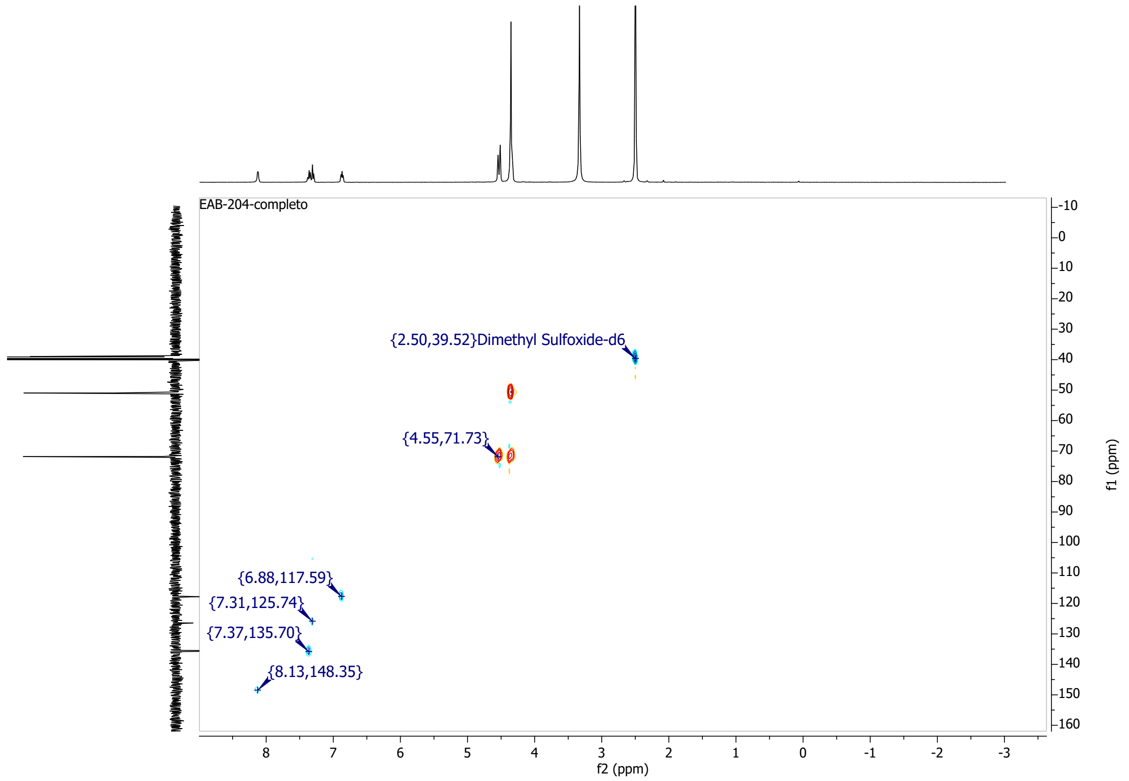

Figure S12. HSQC NMR spectrum of complex **4**.

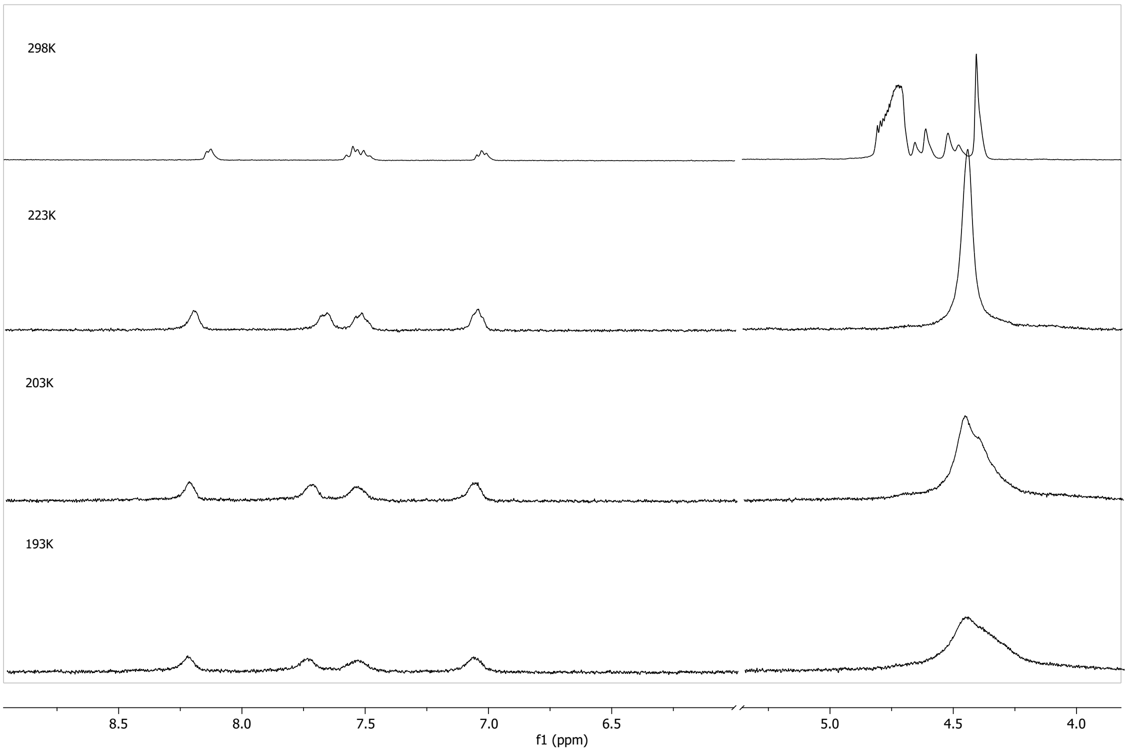

Figure S13. ^{1}H NMR spectra of **3** in MeOD at different temperatures.

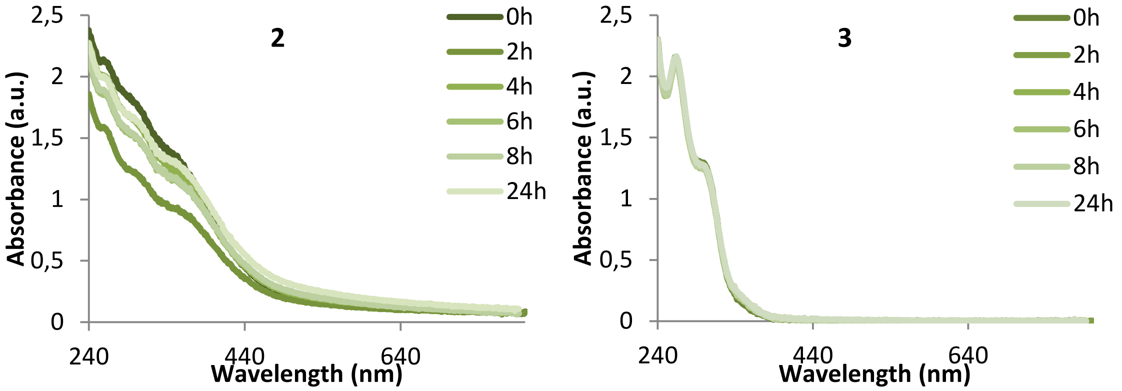

Figure S14. UV-Vis spectra of the heteronuclear complexes **2** and **3** over 24 hours.

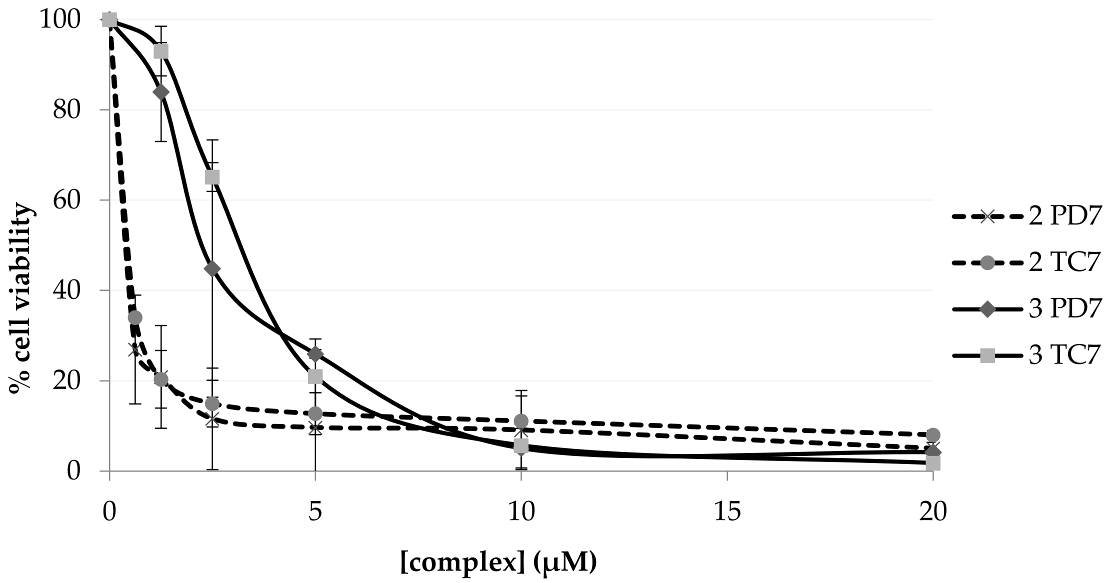

Figure S15. % Cell viability-concentration (μM) plot for the treatment of Caco-2 cells (clones PD7 and TC7) with complexes **2** and **3**

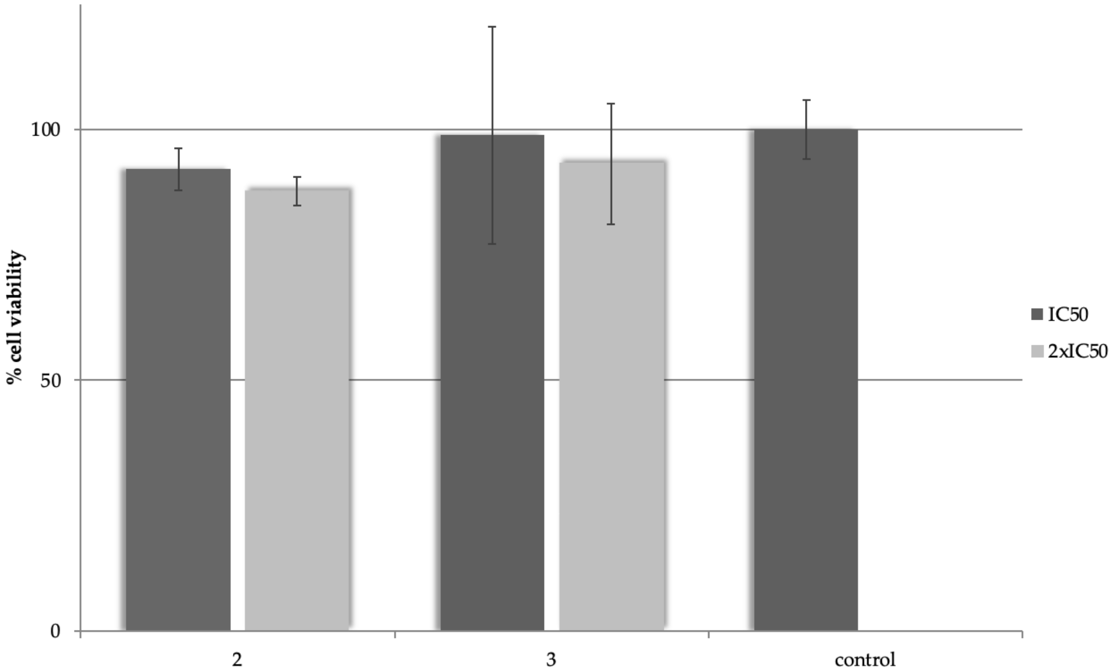

Figure S16. Cell viability (%) of differentiated Caco-2 cells. 92.14 ± 4.22 (complex **2** at IC_{50}); 87.66 ± 2.85(complex **2** at $2xIC_{50}$); 98.87 ± 21.63(complex **3** at IC_{50}), 93.18 ± 11.99 (complex **3** at $2xIC_{50}$).

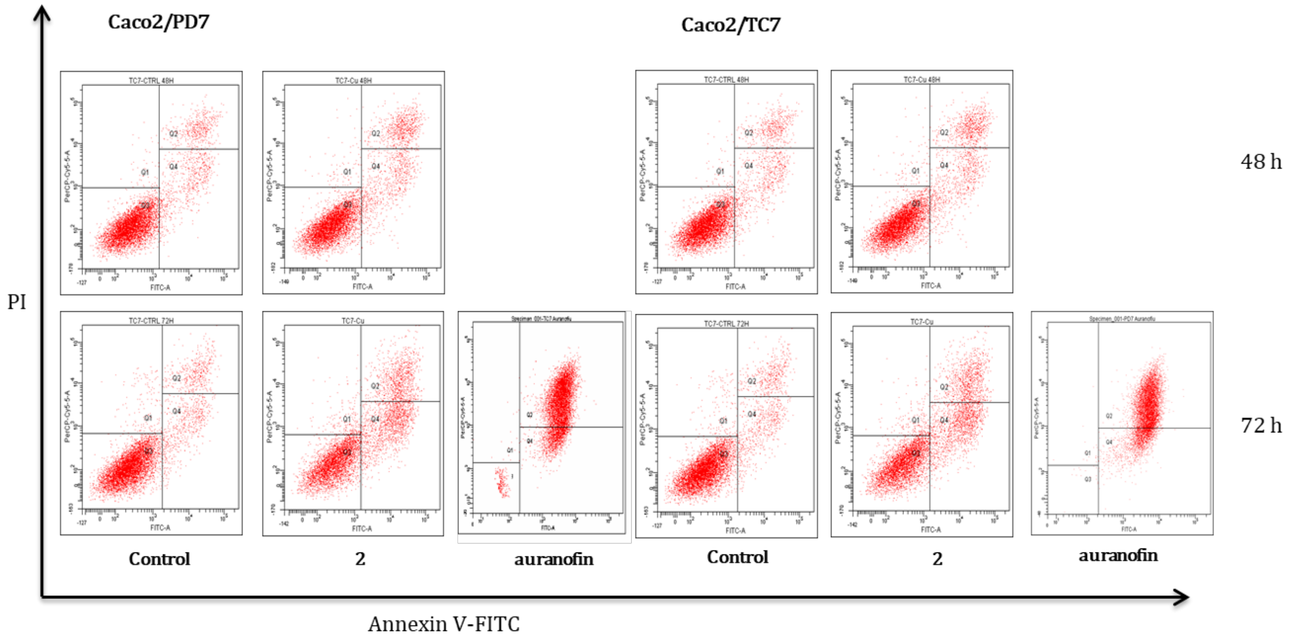

Figure S17. Density plots were obtained from the analysis of Caco-2/TC7 and Caco-2/PD7 cells. Control: non-treated cells; [Cu{Au(Spy)(PTA)}$_2$]PF_6 (**2**) and auranofin as reference drug.

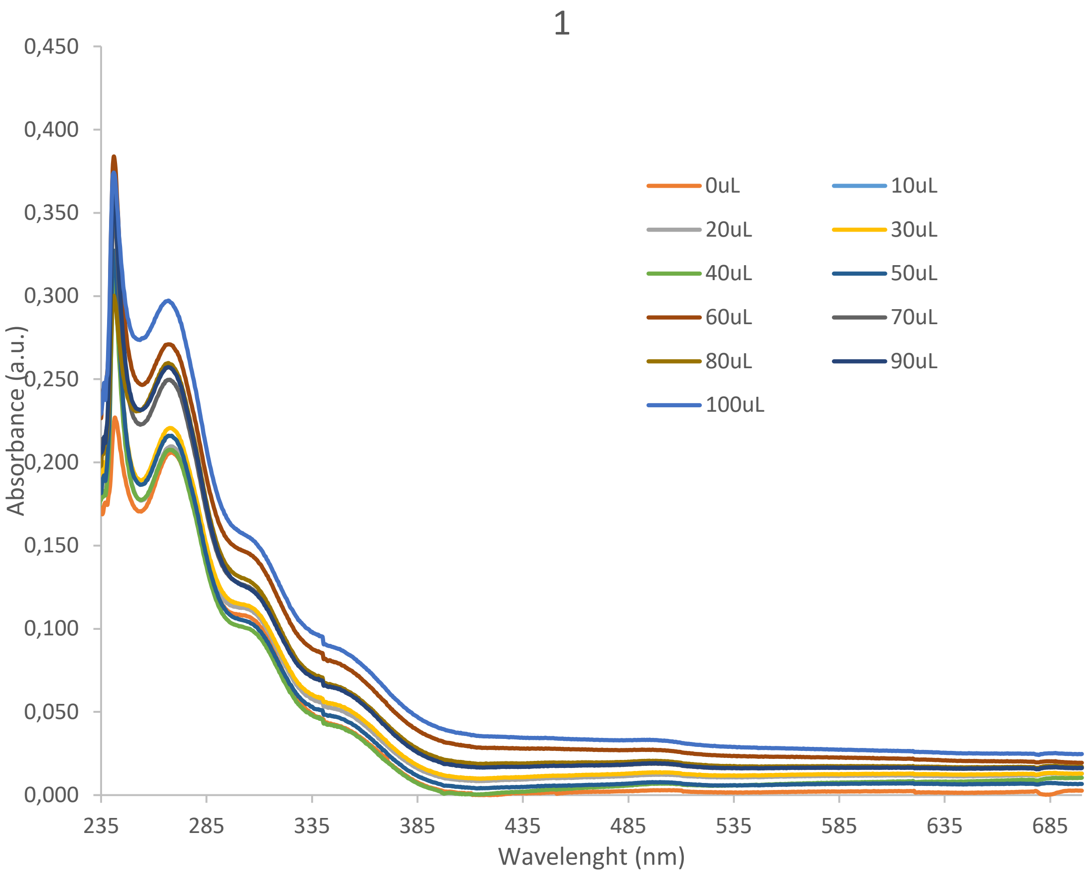

Figure S18. Electronic absorption spectra of complex **1** (20 μM) in Tris-HCl buffer (pH = 7.20) in the absence and the presence of increasing amounts of ct-DNA ([DNA] from 0 to 100 μM).

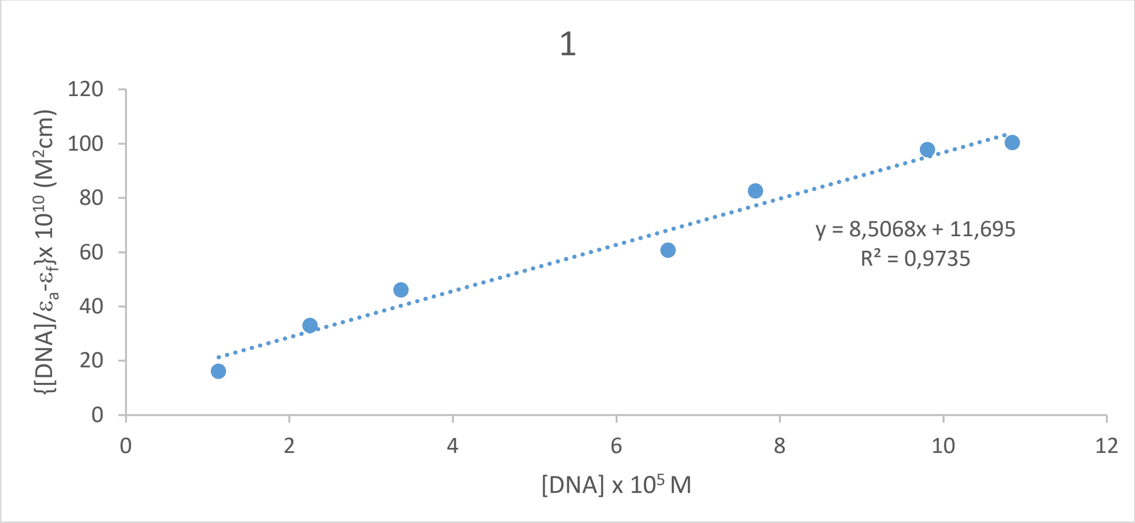

Figure S19. Plot of [DNA]/[εa − εf] *vs*. [DNA] for the titration of ct-DNA with complex **1.**

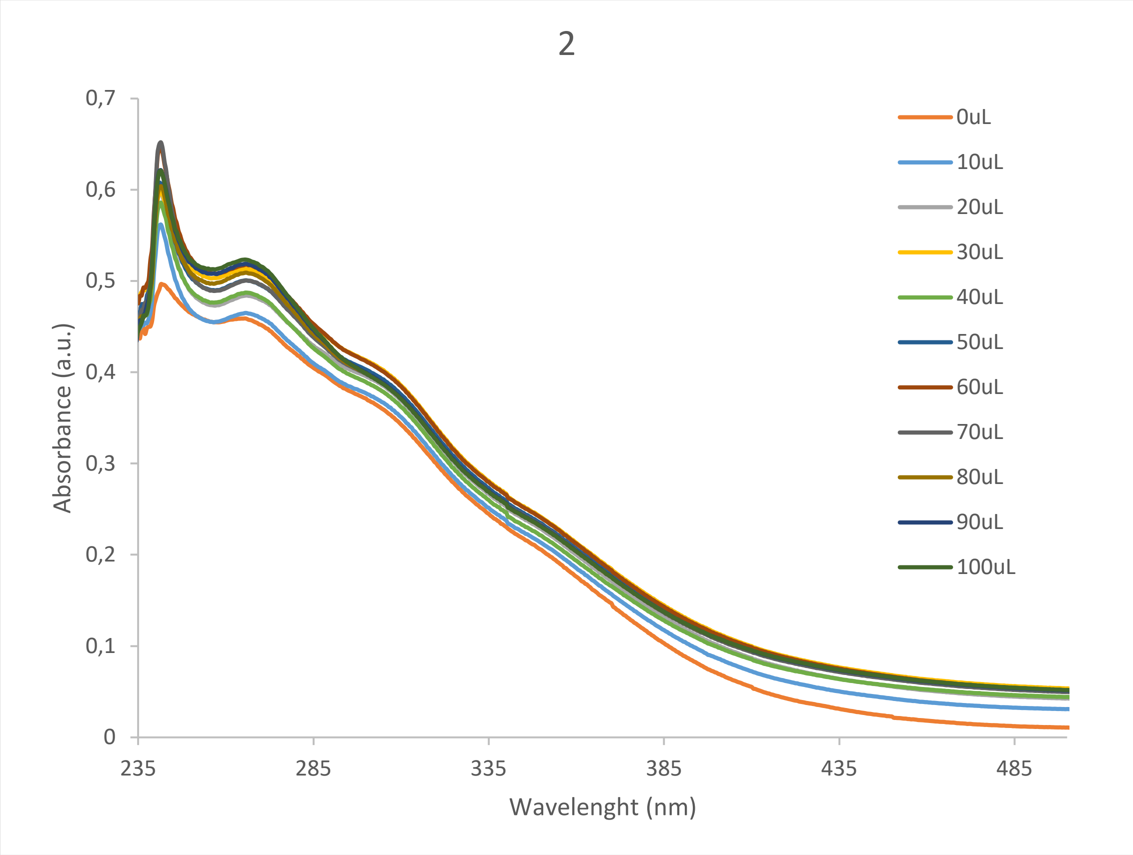

Figure S20. Electronic absorption spectra of complex **2** (20 μM) in Tris-HCl buffer (pH = 7.20) in the absence and the presence of increasing amounts of ct-DNA ([DNA] from 0 to 100 μM).

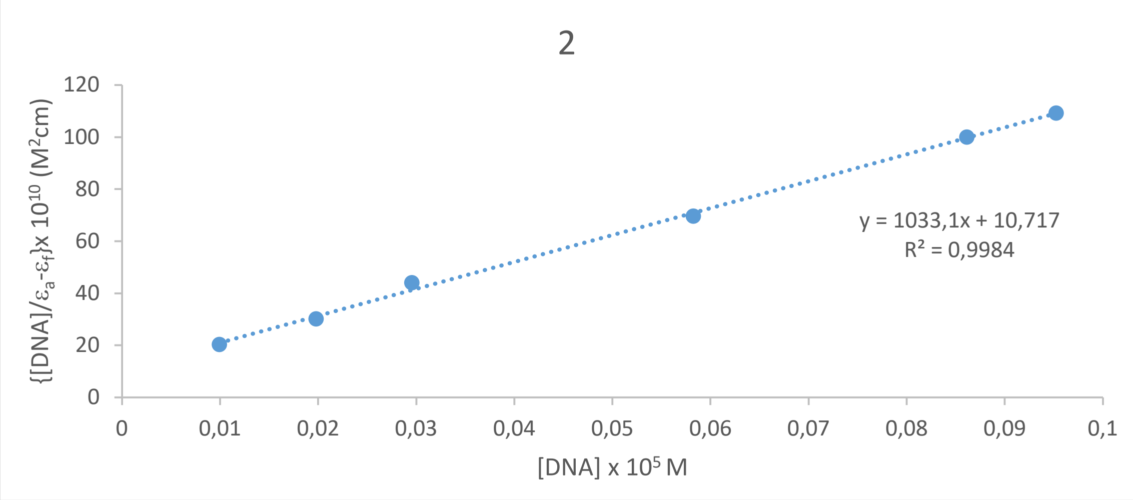

Figure S21. Plot of [DNA]/[εa − εf] *vs*. [DNA] for the titration of ct-DNA with complex **2**

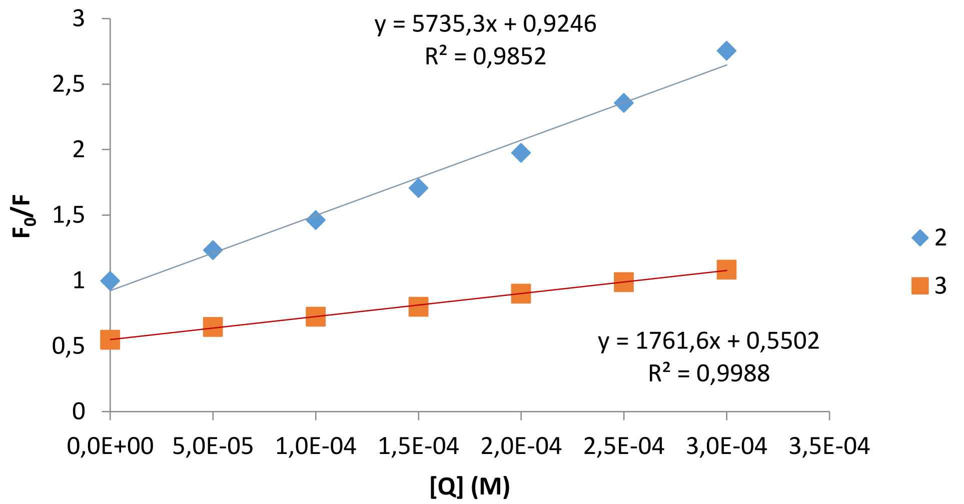

Figure S22. Stern–Volmer plots for the quenching of BSA with increasing amounts of complexes **2** and **3**

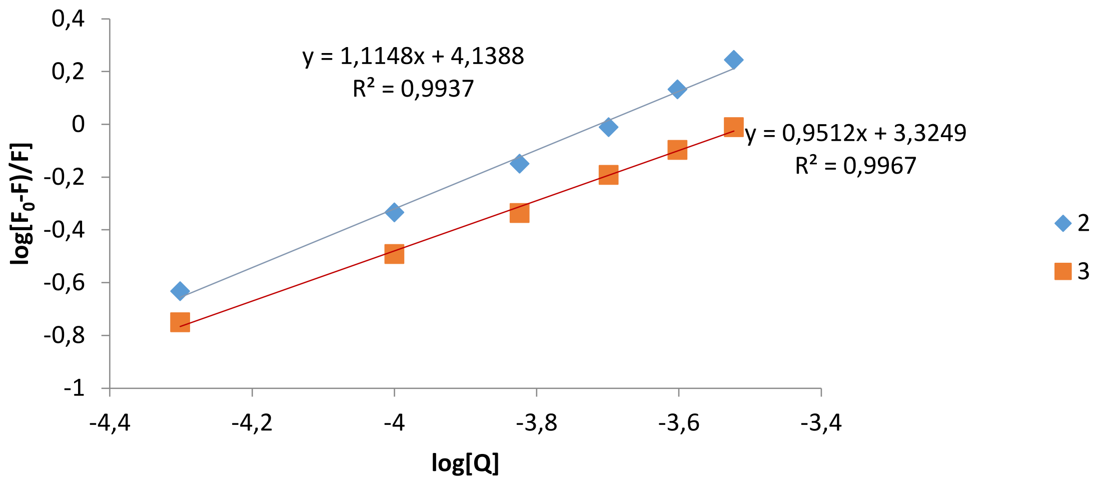

Figure 23. Representation of the modified Stern–Volmer plot. Stern–Volmer equation used: log{(F0 − F)/F} = log Kb + n log[complex] for complexes **2** and **3**.
